# Characterization of the DNA dependent activation of human ARTD2/PARP2

**DOI:** 10.1038/srep34487

**Published:** 2016-10-06

**Authors:** Ezeogo Obaji, Teemu Haikarainen, Lari Lehtiö

**Affiliations:** 1 Faculty of Biochemistry and Molecular Medicine & Biocenter Oulu, University of Oulu, Oulu, Finland

## Abstract

Human ADP-ribosyltransferase 2 (ARTD2/PARP2) is an enzyme catalyzing a post-translational modification, ADP-ribosylation. It is one of the three DNA dependent ARTDs in the 17 member enzyme family. ADP-ribosylation catalyzed by ARTD2 is involved in the regulation of multiple cellular processes such as control of chromatin remodeling, transcription and DNA repair. Here we used a combination of biochemical and biophysical methods to elucidate the structure and function of ARTD2. The solution structures revealed the binding mode of the ARTD2 monomer and dimer to oligonucleotides that mimic damaged DNA. In the complex, DNA binds between the WGR domain and the catalytic fragment. The binding mode is supported by biophysical data that indicate all domains contribute to DNA binding. Also, our study showed that ARTD2 is preferentially activated by short 5′-phosphorylated DNA oligonucleotides. We demonstrate that the N-terminus functions as a high-affinity DNA-binding module, while the WGR domain contributes to DNA binding specificity and subsequent catalytic activation. Our data further suggest that ARTD2 would function in double strand break repair as a dimeric module, while in single strand break repair it would function as a monomer.

ADP-ribosylation is a post-translational modification that was first described in the early 1960s^1^. It is involved in the regulation of numerous cellular processes such as maintenance of genome integrity, chromatin remodeling and transcription control^2^,^3^,^4^,^5^,^6^. The ARTD family in humans consist of 17 enzymes that have been further classified into poly ADP-ribosyltransferases (pARTDs) and mono ADP-ribosyltransferases (mARTDs)^7^,^8^,^9^. pARTDs (ARTD1,2,5,6 and potentially 3 and 4) synthesize polymers of ADP-ribose (PAR) attached to a target protein, while mARTDs (ARTD7–17) only add monomers of ADP-ribose to the target proteins. Two proteins, ARTD9 and ARTD13, are likely inactive. Within the pARTD group, the catalytic activities of ARTD1–3 have been shown to be DNA-damage dependent^2^,^3^,^4^,^5^,^6^. They are activated by DNA breaks, which result in the hydrolysis of nicotinamide adenine dinucleotide (NAD^+^) to nicotinamide and ADP-ribose. The ADP-ribose moiety is successively attached to the acceptor proteins (ARTDs, histones and other DNA repair enzymes) or to a growing PAR chain. PAR adjacent to the DNA breaks forms a scaffold that recruits DNA repair factors^5^,^6^,^10^,^11^.

ARTD1 (PARP1) is the first and best characterized member of the ARTD superfamily and has been shown to play crucial roles in the maintenance of genome stability and in DNA repair^3^,^10^,^12^,^13^. The discovery of ARTD2 (PARP2) was based on the residual DNA-dependent activity observed in ARTD1 deficient mouse embryonic fibroblasts^5^. Similar to ARTD1, ARTD2 interacts with base excision repair (BER)/single strand base excision repair (SSBER) factors, X-ray repair cross complementing 1 (XRCC1), DNA polymerase β, and DNA ligase, which suggests a role in recruiting these factors to the site of DNA damage^3^,^14^,^15^,^16^. ARTD2 can also heterodimerize with ARTD1 leading to their activation^14^. In contrast to ARTD1, ARTD2 lacks the specialized DNA binding zinc fingers in the N-terminus (N), but it has a putative DNA binding motif, which has not been well characterized. It was recently demonstrated that the N-terminus was not strictly required for DNA binding or enzymatic activity^17^, but it was also reported that ARTD2 recognizes apurinic sites through Schiff base formation with the N-terminus^18^. RNA binding activity of the N-terminus has also been reported^19^. The N-terminus is followed by the tryptophan glycine and arginine rich domain (WGR) and the catalytic fragment (CAT) consisting of a regulatory domain (RD) and an ARTD domain (Fig. 1a).

In order to understand the DNA binding and activation of ARTD2, we have used biochemical and biophysical methods to characterize the full length and truncated fragments of the enzyme (Fig. 1a). Our study shows that ARTD2 is activated by various oligonucleotides and high activation of ARTD2 was observed with 10–26 base pair oligonucleotides with a 5′-phosphate. In line with the previous study^17^, we show that the N-terminus is not strictly required for the enzymatic activity as ARTD2_WGR-CAT_ lacking the N-terminus is active although its affinity to DNA is lower. The presence of the N-terminus increases the affinity of all tested DNA molecules (up to 10 nM K_D_) whereas the affinity of constructs lacking the N-terminus was 10–40 folds lower. We also observed that ARTD2 can bind DNA non-specifically through the N-terminus and the binding of ARTD2 to DNA alone is not the switch controlling catalytic activation. We used small-angle X-ray scattering (SAXS) to study solution structures of ARTD2 bound to nicked oligonucleotide mimicking a single stand break (SSB) and to blunt end oligonucleotide mimicking a double strand break (DSB). Biochemical characterization combined with solution structures revealed that the WGR domain is essential for the activation of the enzyme and that the mode of ARTD2 DNA binding and activity could be DNA damage specific.

## Results

### Protein production

**Figure 1:**
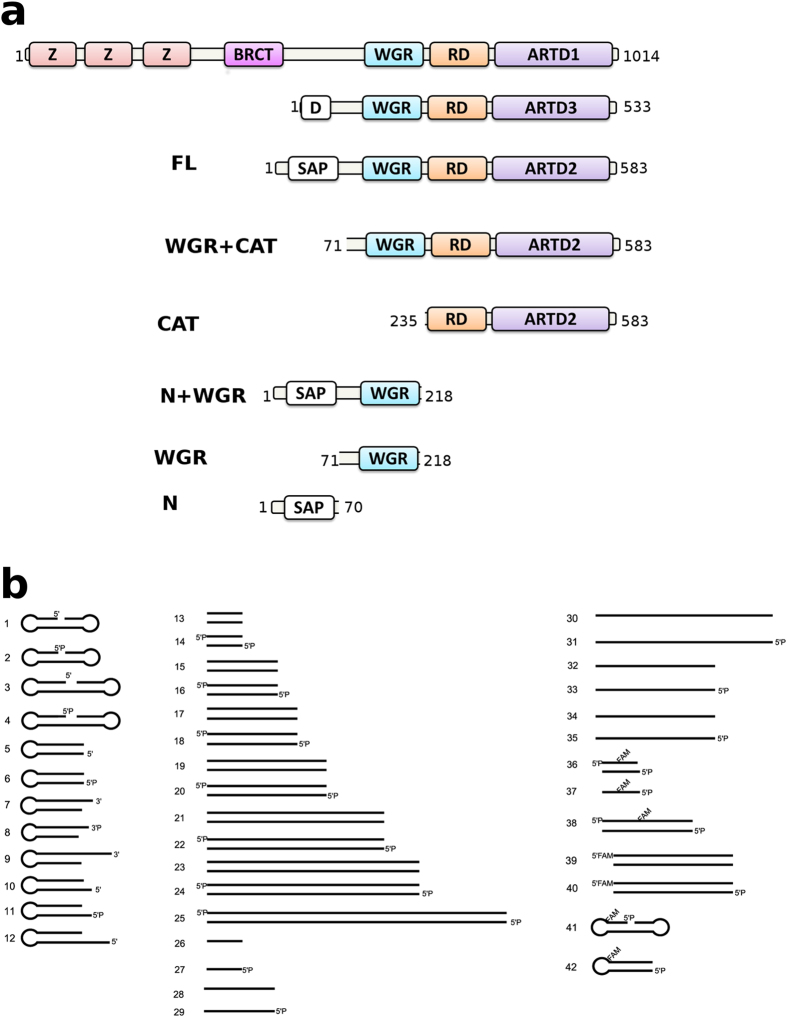
Schematic illustration of DNA dependent ARTDs, ARTD2 constructs and different oligonucleotides used in this study. (**a**) Domain organizations of ARTD1, ARTD2 and ARTD3. ARTD2 constructs used for this study include full length (ARTD2_FL_), N (ARTD2_N_), N+WGR (ARTD2_N+WGR_), WGR (ARTD2_WGR_), WGR+CAT (ARTD2_WGR+CAT_) and CAT (ARTD2_CAT_). (**b**) The oligonucleotides used for the study, which include hairpin, dumbbell shape, palindromic and single stranded DNA. The sequences of the oligonucleotides can be found in Supplementary Table S10.

ARTD2 can be divided into three different structural parts: the N-terminus, WGR domain and the catalytic fragment. In order to study the roles of the different parts, we cloned, expressed and purified the full length ARTD2 and the truncated fragments (Fig. 1a and Supplementary Fig. S1). Protein constructs were all expressed in *E*. *coli* and amounts sufficient for structural and functional studies (4–10 mg/L) were purified. Notably, the DNA contamination of the N-terminal and/or WGR containing protein constructs was removed by heparin affinity chromatography.

### DNA dependent catalytic activity

**Figure 2:**
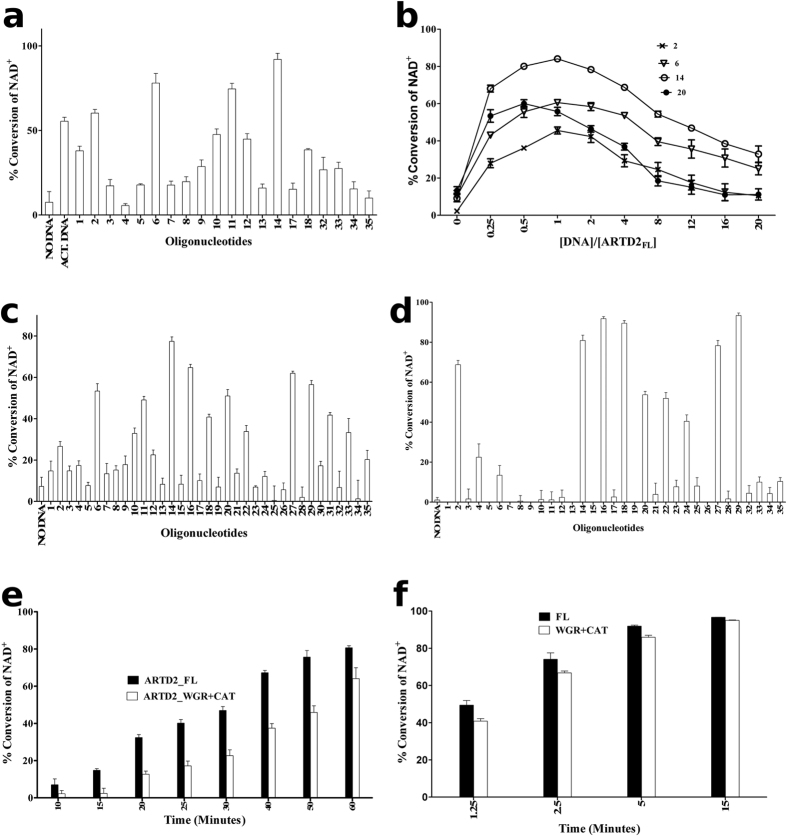
DNA dependent activation of ARTD2. (**a**) Quantification of ARTD2_FL_ catalytic activity with different oligonucleotides at a 10:1 DNA:Protein ratio (500 nM:50 nM, 60 min) in comparison with activated DNA. (**b**) Quantification of the effect of DNA:protein ratio on the activity of ARTD2. DNA concentration was titrated from 0.125 nM to 1 μM using a constant protein concentration of 50 nM. (**c**) Activation of ARTD2_FL_ at a 1:1 protein:DNA ratio without salt (50 nM, 10 min). (**d**) Activation of ARTD2_FL_ at a 1:1 protein:DNA ratio with 150 mM NaCl (100 nM, 30 min). (**e**) Comparison of the DNA dependent activation of the full length enzyme (ARTD2_FL_) against the truncated fragment lacking the N terminus (ARTD2_WGR+CAT_) using 10 nM protein and 10 nM DNA. (**f**) Comparison of the DNA dependent activation of the full length enzyme (ARTD2_FL_) against the truncated fragment lacking the N terminus (ARTD2_WGR+CAT_) using 1 μM protein and 1 μM DNA. Data shown are mean and standard deviation of the experiment carried out in quadruplicate.

Recent reports have indicated that ARTD2 is activated by specific DNA fragments^14^,^16^,^17^,^19^,^20^. It was reported that ARTD2 is selectively activated by 5′-phosphorylated DNA^17^ while other studies have shown that ARTD2 is activated by 5′-overhang DNA and RNA^16^,^19^. We screened different DNA oligonucleotides at a 1:10 protein:DNA ratio using a fluorescence-based activity assay that measures the decrease in the concentration of the substrate NAD^+^ in the reaction. We observed that ARTD2_FL_ is activated by DNase I treated calf thymus DNA and various oligonucleotides (Figs 1b and 2a). ARTD2 was activated by a 38 base long nicked DNA model with a 5′-phosphate (oligonucleotide 2), but not by a similar 48 base long DNA model (oligonucleotide 4). For the blunt ended dumbbell DNA, a 5′-phosphate was required for efficient activation (oligonucleotides 5 and 6). Similarly the 5′-phosphate was preferred for a 5′-overhang (oligonucleotides 10 and 11). The most activating DNA model was a short double stranded DNA with a 5′-phosphate (oligonucleotide 14). Interestingly, an increase in the DNA length decreased the activation of ARTD2 (oligonucleotide 14 vs. 18). Single stranded DNA also activated ARTD2 (oligonucleotide 32–35). Notably, an ARTD inhibitor, 1 μM olaparib, completely inhibited the purified recombinant protein (Supplementary Fig. S2).

Our initial experiments showed that the K_D_ of ARTD2 to DNA would be in the range of 20 nM–50 nM and we expected that saturation of ARTD2 with DNA would result in higher activity. However, when we titrated DNA (12.5 nM–1 μM) against the protein (50 nM ARTD2_FL_) we observed that the highest activity was achieved at a 1:1 or 2:1 ratio of ARTD2 protein to DNA depending on the oligonucleotide (Fig. 2b). Oversaturation of ARTD2 with DNA resulted in a decreased NAD^+^ hydrolysis in all cases. This suggests that automodification would require dimerization of the enzyme at the DNA damage site. Increase in DNA concentration or increase in the possible binding sites at the DNA could result in a non-catalytic 1:1 protein:DNA complexes that would lower the activity. Based on this hypothesis, we rescreened the activation of ARTD2 by the oligonucleotides at a 1:1 Protein-to-DNA ratio and included more oligonucleotides (oligonucleotides 19–31) (Figs 1b and 2c). Increasing salt concentration decreases ARTD2 activity, but we carried out the experiment also in the presence of 150 mM salt using higher protein concentration (50 nM vs. 100 nM) and a longer incubation time (10 min vs. 30 min) (Fig. 2d). Highest activity was achieved with dumbbell DNA molecules (oligonucleotides 6, 10 and 11), double stranded blunt ended DNA molecules (oligonucleotides 14, 16, 18, 20, 22) and single stranded DNA molecules with 5′-phosphates (oligonucleotides 27 and 29) (Fig. 2c,d). A nick DNA model (oligonucleotide 2) was not among the top activating oligonucleotides (Fig. 2c) although its relative activation was comparable in the higher salt conditions (Fig. 2d). Oligonucleotide 8 with a 3′-phosphate did not activate the enzyme in either condition. These results demonstrated that not only is ARTD2 selectively activated by various 5′-phosphorylated DNAs but also the length of the DNA has a significant effect on the activity *in vitro*. In both cases the results showed clearly that ARTD2 activation is strongly dependent on the DNA length (Fig. 2c,d) as an increase in the DNA length resulted in decreased ARTD2 catalytic activity.

### Contribution of the N-terminus to the catalytic function

In order to provide an explanation for the role of the N-terminus in the catalytic activity of ARTD2, we compared the rate of activation of ARTD2_FL_ with ARTD2_WGR+CAT_ using the most activating DNA (oligonucleotide 14). We observed that the rate of activation of ARTD2_FL_ is significantly higher than that of ARTD2_WGR+CAT_ (Fig. 2e). In this low DNA and protein concentration (10 nM), this difference could be the result of lower DNA binding affinity of ARTD2_WGR+CAT_. Therefore the rate of activation at 1 μM protein and DNA concentrations was measured and it was observed that the N-terminus is not required for the catalytic activation of ARTD2 at high protein and DNA concentrations (Fig. 2f).

### DNA binding properties of ARTD2

**Figure 3:**
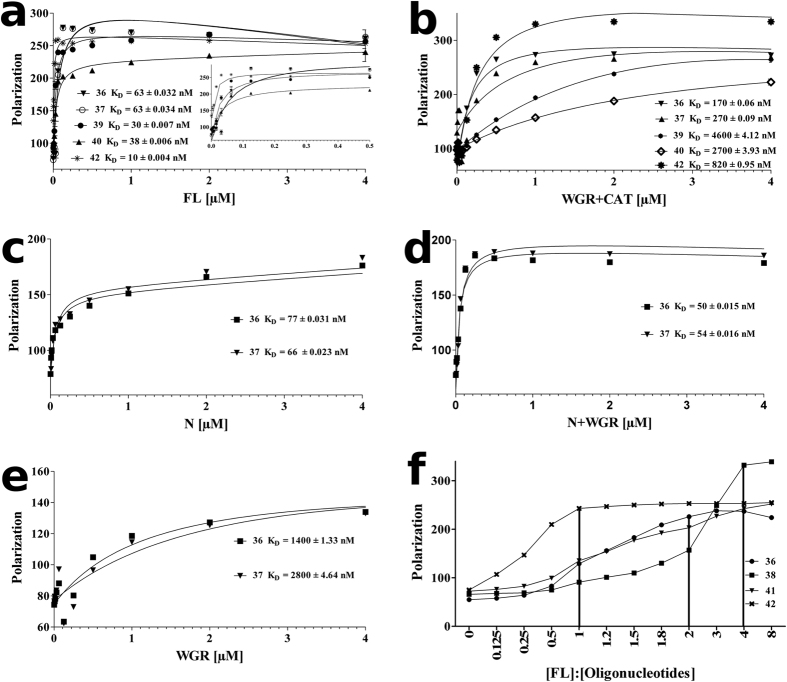
Fluorescence polarization assay showing the binding affinity of ARTD2 domains to different oligonucleotides. The oligonucleotides used are 36, 37, 38 39, 40, 41 and 42 (Fig. 1b). (**a**–**e**) Affinities of ARTD2_FL_, ARTD2_WGR+CAT_, ARTD2_N,_ ARTD2_N+WGR_ and ARTD2_WGR_ to the oligonucleotides. (**f**) Stoichiometry analysis of ARTD2_FL_ binding to oligonucleotides 36, 38, 41 and 42. The saturation point was considered as the stoichiometric ratio. Data shown are mean and standard deviation of the experiment carried out in triplicate.

The binding affinity of ARTD2 (full length and the different domains) to selected DNA molecules was studied using a fluorescence polarization assay (FP). The affinity of ARTD2_FL_ towards activating and fluorescence labeled oligonucleotides was between 10 and 63 nM (Fig. 3a). The affinity of the construct lacking the N-terminus was significantly lower with a K_D_ up to 4 μM (Fig. 3b). Oligonucleotide 14 had the highest activating property in the assays and the binding affinity of ARTD2_WGR+CAT_ to the corresponding fluorescently tagged oligonucleotide (oligonucleotide 36) was the highest (170 nM) of the oligonucleotides tested. Notably, with ARTD2_FL_ the binding affinity is independent of the presence of 5′-phosphorylation. This was observed with oligonucleotides 39 and 40, which had similar affinities for the enzyme. ARTD2_N_ and ARTD2_N+WGR_ had affinities in the same range as ARTD2_FL_ (Fig. 3c,d), whereas ARTD2_WGR_ had a K_D_ in the micromolar range to the activating single and double stranded oligonucleotides (Fig. 3e). It appears that while the N-terminus functions as a non-specific, high-affinity DNA-binding module, the WGR domain is a low-affinity, selective DNA-binder.

**Figure 4:**
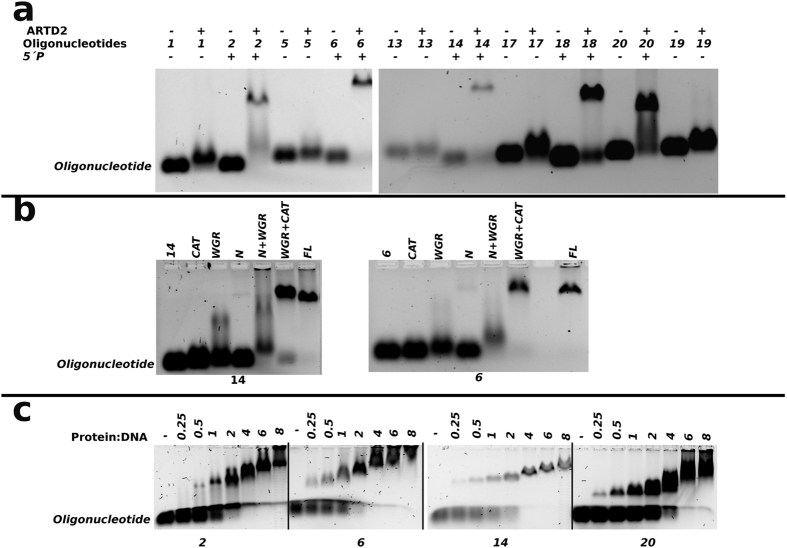
EMSA assay of ARTD2 domains showing the binding and stoichiometry to different oligonucleotides. (**a**) The binding of ARTD2_FL_ (1 μM) to different DNA oligonucleotides (1 μM). A supershift was observed with the 5′-phosphorylated DNA oligonucleotides. (**b**) Binding of ARTD2_FL_ and the truncated fragments to oligonucleotides 6 and 14. (**c**) Stoichiometry analysis of ARTD2_FL_ binding to oligonucleotide 2 (equivalent of oligonucleotide 41), oligonucleotide 6 (equivalent of oligonucleotide 42), oligonucleotide 14 (equivalent of oligonucleotide 36) and oligonucleotide 18 (equivalent of oligonucleotide 38). The protein:DNA ratio used was 0–8 and DNA was kept constant at 1 μM.

We also studied the binding of ARTD2 to the activating and non-activating oligonucleotides using EMSA (Fig. 4a,b). We observed that ARTD2_FL_ upon binding to the 5′-phosphorylated oligonucleotide resulted in a clear supershift while nonphosphorylated oligonucleotides did not show a clear supershift (Fig. 4a). As an example, nonphosphorylated oligonucleotides 1, 5, 13, 17 and 19 did not bind efficiently to ARTD2, whereas similar phosphorylated oligonucleotides 2, 6, 14, 18 and 20 resulted in a supershift that indicated binding. The binding of ARTD2_FL_ in comparison with the truncated ARTD2 constructs was also studied using oligonucleotides 6 and 14. As expected, ARTD_FL_ caused a supershift on the gel and ARTD2_CAT_ did not seem to bind DNA (Fig. 4b). Binding of the ARTD2_WGR+CAT_ to the oligonucleotides resulted in the same super shift on the gel as with the full-length enzyme. ARTD2_N_ did not cause a supershift despite the high affinity measured in FP, but the ARTD_WGR_ domain caused a smear on the gel with oligonucleotide 14 that indicated binding. Interestingly, ARTD2_N+WGR_ caused even more delay in the DNA on the gel. The results indicate that the N-terminus has a high affinity for DNA, but the off rate is likely also high explaining why there is no observable supershift in EMSA. The K_D_ of the WGR domain to DNA is lower, but the binding is more specific and the off rate is lower, which contributes to the observable supershift with ARTD2_WGR_ (Fig. 4b). Notably, a 5′-phosphate is required for the specific binding with the WGR domain. The catalytic fragment also clearly affects the DNA binding indirectly through the other domains as ARTD2_WGR+CAT_ and ARTD2_FL_ cause a supershift, whereas ARTD2_N+WGR_ only causes a slight delay of DNA on the gel (Fig. 4b).

### ARTD2 binds to DNA with varying stoichiometries

We studied the stoichiometry of the DNA oligonucleotide ARTD2_FL_ complexes using an FP assay where a DNA concentration (250 nM) well above the K_D_ was used and titrated with protein. Double stranded palindromic 5′-phosphate (oligonucleotides 36 and 38), dumbbell blunt end 5′-phosphate (oligonucleotide 42) and nicked 5′-phosphate DNAs (oligonucleotide 41) were used. ARTD2 was observed to bind to oligonucleotide 42 as a monomer and to oligonucleotide 36 as a dimer (Fig. 3f). With oligonucleotides 38 and 41, different stoichiometries were observed (ranging from 1:1 to 4:1 protein-to-DNA).

Furthermore, we titrated the protein concentration from 0.25–8 μM against 1 μM DNA and analyzed the samples using an electrophoretic mobility shift assay (EMSA). In the EMSA assay there was a continuous increase in the band shift with increasing protein concentration. With oligonucleotides 6 and 14 (the equivalent of fluorescein tagged oligonucleotides 42 and 36) there was no free oligonucleotide at a stoichiometric ratio higher than 2:1 protein to DNA ratio (Fig. 4c). In contrast, with oligonucleotide 18 (equivalent of fluorescein tagged oligonucleotide 38) there was no free oligonucleotide only after a 4:1 protein-to-DNA ratio. With oligonucleotide 2 (equivalent of fluorescein tagged oligonucleotide 41) there was still some free oligonucleotide even at a protein-to-DNA ratio of 4:1 (Fig. 4c).

### Solution structures of ARTD2

**Figure 5:**
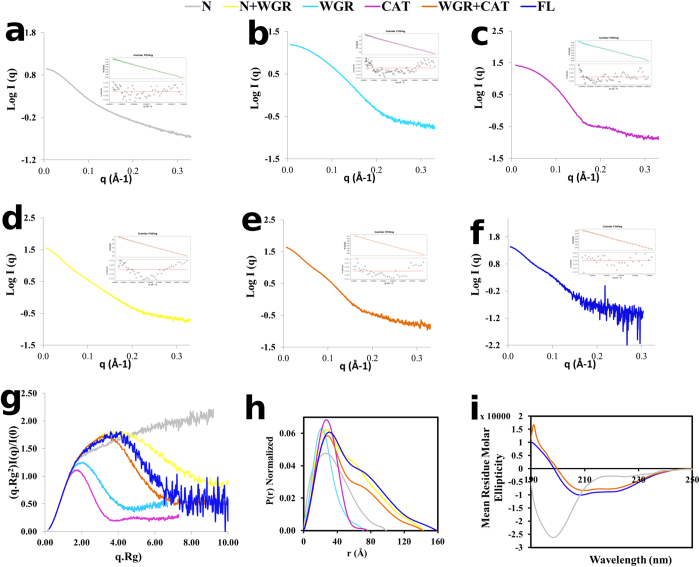
Characterization of ARTD2_FL_ and the truncated fragments using small-angle X-ray scattering. (**a**–**f**) SAXS data plots as well as the Guinier plots of ARTD2_N_, ARTD2_WGR_, ARTD2_CAT_, ARTD2_N+WGR_, ARTD2_WGR+CAT_, and ARTD2_FL_, respectively. The zoom in view of the Guinier region is provided in Supplementary Fig. S3. (**g**) The normalized Krakty plot analysis of ARTD2_FL_ and the truncated fragments. (**h**) The P(r) distribution profile of ARTD2_FL_ and the truncated fragments. (**i**) Secondary structure analysis of ARTD2_FL_, ARTD2_N_ and ARTD2_WGR+CAT_ with CD.

The structure of the human ARTD2 catalytic domain (ARTD2_CAT_) in complex with inhibitors has been solved using X-ray crystallography^21^,^22^. At the moment, there is no high-resolution structure of the other domains or the full-length enzyme. Here we have used SAXS to provide a low-resolution structure of the full-length enzyme and the truncated fragments. The scattering curves and Guinier plots for all the proteins were generated after subtraction of the buffer contribution (Fig. 5a–f and Supplementary Fig. S3). From the Kratky plot analysis of the individual domains, we observed that ARTD2_N_ exhibits behavior typical of a disordered molecule while the other domains, especially ARTD2_CAT_ and ARTD2_WGR_, exhibit behavior typical of a globular molecule with one distinct peak (Fig. 5g). From the processed scattering intensity, the radius of gyration (R_g_), molecular weight (MW), maximum dimension (D_max_), and volume (porod volume) were derived (Tables 1 and 2). With the exception of ARTD2_N+WGR_, the experimental MWs of all the fragments are in good correlation with the calculated monomeric MWs. The experimental MW of the ARTD2_N+WGR_ (47.7 kDa) indicates that it forms a dimer in solution. This correlates with the retention volume (56 mL) observed during size exclusion purification, when compared with e.g. monomeric CAT fragment with MW of 39.8 kDa (retention volume 61 mL). The D_max_ and R_g_ of the ARTD2_N_ are higher than expected, which agrees with the fact that it is highly disordered (Tables 1 and 2, Fig. 5a). The pair distribution function [P(r)] curve of ARTD2_FL_ and ARTD2_WGR+CAT_ has a dumbbell shape containing more than one maximum, which is typical for molecules with individual domains connected by flexible linkers (Fig. 5h). This supports the structural organization of ARTD2_FL_ having two domains (CAT and WGR), whereas the N-terminus does not form a folded domain. The P(r) plot for ARTD2_N+WGR_ shows a similar dumbbell shape, which is due to the dimeric form of the construct. The P(r) functions of ARTD2_N_, ARTD2_WGR_ and ARTD2_CAT_ are characterized with a single maximum indicating that they consist of one structural domain (Fig. 5h). The experimental SAXS data from the CAT domain match the scattering intensity of the crystal structure with chi^2^ of 1.26 (Supplementary Fig. S4). From the SAXS data we generated *ab initio* models of each of the proteins (Supplementary Fig. S5). These molecular shapes agree with above analysis of the P(r) functions.

The SAXS study indicated that the N-terminus is structurally disordered. To provide further evidence, we measured circular dichroism spectra of the protein and compared it with spectra of ARTD2_FL_ and ARTD2_WGR+CAT_. As expected, the CD spectrum of ARTD2_N_ is characterized with a single minimum at a wavelength of around 200 nm, whereas with ARTD2_FL_ and ARTD_WGR+CAT_ the spectra represent typical folded molecules with secondary structures (Fig. 5i). The single minimum of ARTD2_N_ at 200 nm showed that the N-terminus of ARTD2 is structurally disordered and is completely in agreement with a previous report^20^.

### Solution structures of ARTD2_-_DNA complexes

We have shown that ARTD2 is preferentially activated by 10–26 base pair oligonucleotides with a 5′-phosphate (Fig. 2c,d). From the FP analysis we have shown that ARTD2 binds to the different activating oligonucleotides as a monomer, dimer and other higher oligomeric states depending on the DNA structure (Fig. 3f). Prior to the SAXS studies, HPLC analysis of ARTD2_FL_ and ARTD2_WGR+CAT_ in complex with oligonucleotides 2, 6 or 14 (FP equivalents 41, 42 and 36 respectively) showed that upon DNA binding ARTD2 adopts different oligomeric states like in the FP stoichiometry analysis (Fig. 3f and Supplementary Fig. S6). Notably, with oligonucleotide 2, there were different oligomeric states and the major peak was the 1:1 protein DNA complex (Supplementary Fig. S6). With oligonucleotides 6 and 14, the major peak was the 2:1 protein DNA complexes (Supplementary Fig. S6).

**Figure 6:**
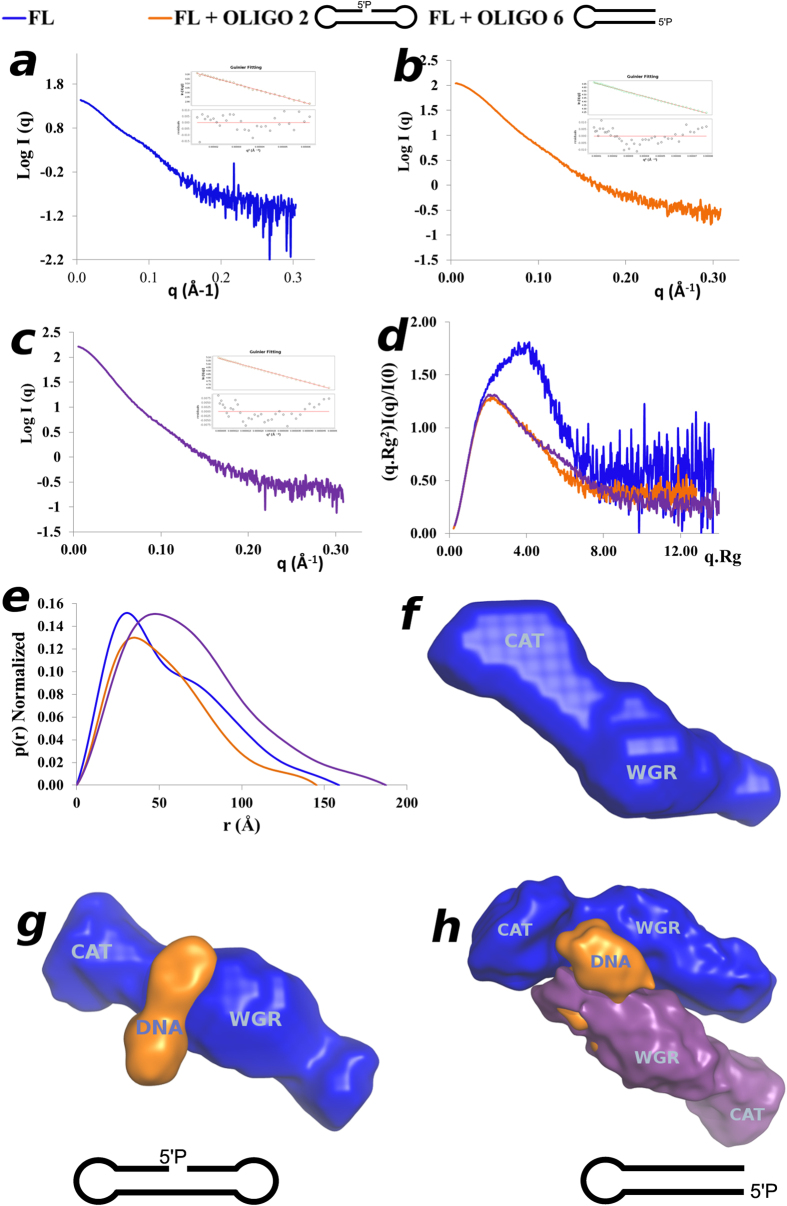
Characterization of ARTD2_FL_ and its DNA complexes using small angle X-ray scattering. (**a**–**c**) SAXS data plots as well as the Guinier plots of ARTD2_FL_, ARTD2 in complex with Oligonucleotide 2 and 6, respectively. (**d**) Kratky plot analysis of ARTD2_FL_ and its complex with oligonucleotides 2 and 6. (**e**) P(r) distribution profile of ARTD2_FL_ and its complex with DNA oligonucleotides 2 and 6. (**f**) The *Ab initio* model of ARTD2_FL_ was reconstructed using DAMMIF and shown is the averaged filtered shape from DAMFILT (**f**,**g**) ARTD2-DNA complexes (nicked and blunt end oligonucleotide 2 and 6, respectively) were reconstituted using MONSA and shown is the averaged filtered shape from DAMFILT.

Based on the observed property of ARTD2 to form different oligomeric states upon DNA binding, it was necessary to collect the SAXS data of the DNA complexes using an on-line HPLC system. SAXS data were measured for oligonucleotides 2 and 6. In agreement with the HPLC studies prior to the SAXS studies, there were different oligomeric states based on the different chromatograms (Supplementary Fig. S6). With oligonucleotide 2 (nicked DNA) the major monomeric complex and with oligonucleotide 6 (short double stranded dumbbell DNA) the major dimeric complex were used for the subsequent data analysis. Also the Rg profiles across the targeted peaks show that they are highly monodispersed (Supplementary Fig. S6). The data of the higher oligomeric complexes could not be processed due to high polydispersity and low scattering intensity (Supplementary Fig. S6). The scattering curves and Guinier plots after subtraction of the background signals are shown in Fig. 6a–c and Supplementary Fig. S3. From the Kratky plots, the apo protein as well as the complexes show curves typical for globular molecules (Fig. 6d). The Kratky plots indicate that upon DNA binding ARTD2 assumes a more compact structure. In agreement with the Krakty plots, the P(r) analysis clearly shows that upon binding to DNA, ARTD2 assumes a compact structure without double maxima as was observed in the absence of DNA (Figs 5h and 6e). With oligonucleotide 2, the D_max_ and R_g_ were smaller in comparison to ARTD_FL_, which also indicates a more compact structure (Tables 1 and 2). With oligonucleotide 6, the D_max_ and R_g_ were observed to be higher than those of the apo enzyme as well as its complex with oligonucleotide 2, which indicates dimerization of the enzyme.

The average filtered *ab initio* models generated with MONSA^23^ show the overall structures of the ARTD_FL_ with and without the oligonucleotides (Fig. 6f–h). In the 1:1 complex with the nicked oligonucleotide, ARTD2 interacts with the DNA through a middle part corresponding to a WGR domain (Fig. 6g), where the assignment of the domains in the DNA complexes is based on rigid body modelling done on the apo enzyme (Supplementary Fig. S4). In case of a blunt end dumbbell, the ARTD2 forms a dimer around the oligonucleotide (Fig. 6h). In the model it appears that the catalytic domains are facing in opposite directions and the interaction with the DNA would not be identical for both monomers. The *ab initio* models give information on the average structures in solution. Considering that oligonucleotide 6 is one of the most activating DNAs, we expect that this may be an ideal binding mode in solution allowing one protein to ADP-ribosylate the other. It also provides an explanation to why we observed higher activity with short blunt end DNA molecules than with the nicked DNA molecules.

## Discussion

The role of ARTD2 in base excision repair/single strand base excision repair (BER/SSBER) and homologous recombination has been a subject of discussion. The importance of ARTD2 in BER/SSBER and double strand breaks is based on its interaction with the main DNA repair factors, a function similar to that reported for ARTD1^3^,^14^,^24^,^25^,^26^,^27^. Despite the recent data on ARTD2 DNA binding and activation^17^,^20^, is still not clear how the enzyme is activated by many different forms of DNA. We studied activation, affinity, stoichiometry, oligomerization and solution structures of the enzyme upon binding to different DNA molecules to gain insight into the specificity of the DNA damage recognition mechanism of ARTD2. SAXS structures provide the first experimental views of ARTD2-DNA complexes.

Our results show that ARTD2 is robustly activated when the DNA is phosphorylated at the 5′-end, which is in agreement with the previous reports^17^,^20^. Interestingly the oligonucleotide identified by Langelier *et al*. as the most activating DNA model was a very weak activator of ARTD2 in our assays (oligonucleotide 4, Fig. 2a,c,d). This could result from the use of slightly different experimental conditions and assays. It could also depend for example on the isoform used in the studies. Langelier *et al*. used ARTD2 isoform 2 (NP_001036083), which is a shorter isoform of the enzyme, whereas we used the canonical ARTD2 isoform (NP_005475). Isoform 2 lacks 13 internal amino acid residues (G68-S80) (Supplementary Fig. S7). In contrast to the earlier report, we also removed the N-terminal histidine-tag from the recombinant enzyme. Despite the differences, ARTD2 was consistently more activated by the oligonucleotides containing 5′-phosphate also in our assays (Fig. 2a,c). The activation of ARTD2 by single stranded RNA with 5′-phosphate is comparable to the activation by single stranded DNA (Supplementary Fig. S8). Importantly, the presence of 5′-phosphate on the short DNA molecules results in robust catalytic activation, whereas we did not observe activation of ARTD2 by the PAR chains (Supplementary Fig. S8).

In our study, we discovered that the length of the oligonucleotide and increase in oligonucleotide concentration had negative effects on the activation of ARTD2. With 10–18 base pair DNA ARTD2 was observed to have the highest activity at a ratio of 1:1 and 2:1 protein:DNA complex, while with 26–33 base pair DNA, the highest activity was at a ratio of 2:1 and 4:1 protein:DNA complex (Fig. 2b). Also oligonucleotides of more than 50 base pairs were observed to have a negative effect on the activation of ARTD2 (Fig. 2c,d). Based on this, we hypothesize that in the presence of excessive DNA damage, more ARTD2 binds to the lesion site as monomers, which are not capable of robust automodification. In the shorter double stranded oligonucleotide, there is effectively only one possible binding site similar to a dumbbell oligonucleotide, which increases the protein:DNA damage site ratio. The nicked DNA would be expected to activate the enzyme similar to the short oligonucleotides but that was not observed as the activity with the nicked DNA was lower (Fig. 2a,c). The mechanism employed by the enzyme could be different in this case and binding to the nick could help hetero-oligomerization with different protein targets. Therefore, with an exposed DNA end, a dimer of ARTD2 is efficiently formed and automodification is efficient at least *in vitro*. We observed that high salt concentration lowers the activity of ARTD2 roughly 6-fold as similar NAD^+^ conversion required more protein and longer incubation time. This effect could be related to disruption of protein-protein interaction needed at the DNA blunt ends. At the high salt conditions, although the overall activity is lower, activation by phosphorylated nick and double stranded DNA were comparable (Fig. 2d). Monomer dimer equilibrium could provide a possible mechanism for ARTD2 to regulate various DNA repair pathways, although the possible binding partners and environment at the chromosome will differ from the *in vitro* enzymatic assays carried out in this study.

Notably, the affinity of ARTD2 towards the oligonucleotides was independent of whether they are activating or not (Figs 2 and 3). The affinity of the full-length enzyme in comparison to ARTD2_N_ and ARTD2_N+WGR_ was observed to be within the same range (Fig. 3), highlighting the roles of the N-terminus and WGR domain as the main DNA binding modules. The N-terminus interacts with DNA and contributes to the high binding affinity of the enzyme to the oligonucleotide. The WGR domain contributes to the DNA binding of the enzyme as well as to the catalytic activation and may be an essential factor for the recognition of the 5′-phosphate on the oligonucleotides. Also the catalytic fragment is important in the binding of the enzyme to the activating DNA molecules (Figs 3b,e and 4b). Interestingly, the N-terminus is not required for the catalytic activity in high protein-DNA concentrations, but appears to be necessary when concentrations are low (Fig. 2e,f).

We also show that the N-terminus of ARTD2 is highly disordered (Fig. 5g,i) and this is inline with a previous report^20^. The high affinity of the N-terminus observed in FP experiments (Fig. 3) could enable ARTD2 to scan the DNA whereas the WGR domain would act as the damage detection module as it forms a stable complex with the DNA in EMSA (Fig. 4b). It may not only be the WGR domain that contributes to the damage site detection, but also the catalytic fragment could contribute to the DNA binding (Fig. 4b). SAXS models show that the main interface interacting with the DNA is in the middle of the protein, which would be the region between the WGR domain and the catalytic fragment (Fig. 6g,h). It would suggest that when ARTD2 recognizes a DNA damage site, a structural change in the complex would relocate the N-terminus from the DNA break. The contribution of the catalytic fragment to the DNA binding is evident in the FP and the EMSA experiments and it can be visualized with the experimental low-resolution model (Figs 3b,e, 4b and 6g).

Solution structures show that ARTD2 can bind DNA as a monomer and as a dimer. To SSB ARTD2 binds preferentially as a monomer, while to DSB it binds mostly as a dimer. In case of a dimer DNA in the SAXS envelope (Fig. 6h) does not bind symmetrically to both ARTD2 monomers and this indicates that only one ARTD2 interacts productively with the DNA resulting in activation and consequent trans-automodification as reported with ARTD1^28^. Similar to the 1:1 complex, DNA would bind close to the catalytic part possibly contributing to the increased stability of the complex observed in EMSA (Figs 4b and 6h). N-terminal lysines (36 and 37) have been identified as ADP-ribosylation sites in ARTD2^29^ and in agreement with this the N-terminal region is located close to the catalytic site of an adjacent ARTD2 in the SAXS model. It has been reported that WGR+CAT domains of ARTD1-3 tightly cooperate with their corresponding N-terminal region^30^. Other modification sites within the WGR and catalytic domains of ARTD2 have also been identified^31^. The most striking feature of the solution structure is how ARTD2 preferentially binds to nicked DNA (mimicking single strand DNA break) as a monomer and to blunt end DNA (mimicking double strand DNA break) as a dimer. Notably the binding mode of ARTD2 to DNA may differ from that of ARTD1, which detects the DNA damage through multiple zinc fingers^32^ which are not present in ARTD2.

Collectively, the N-terminal region of ARTD2 is highly disordered and has the ability to interact non-specifically with DNA. The WGR domain is an important DNA damage recognition site and is essential for the DNA dependent activity of ARTD2. ARTD2 is preferentially activated by short 5′-phosphoryated oligonucleotides and the structure and length of the DNA is very important for the robust activation of the enzyme *in vitro*. The binding ratio of ARTD2 to nicked DNA (mimicking single strand DNA break) is mostly one protein to one DNA while to blunt end DNA (mimicking double strand DNA break) two protein to one DNA. This study highlights the different binding modes employed by ARTD2, which could enable it to function in multiple DNA repair pathways.

## Materials and Methods

### Cloning

The codon optimized (*E*. *coli*) cDNA of the human full length ARTD2 isoform 1 (NP_005475) was purchased from Genescript. Full-length ARTD2 as well as ARTD2_N+WGR_ and ARTD2_N_ were cloned by PCR extension cloning into a pNH-trxt vector containing an N-terminal 6xHis-tag, a thioredoxin fusion protein and a TEV-protease cleavage site (marked with *) (MHHHHHHSSGMSDKIIHLTDDSFDTDVLKADGAILVDFWAEWCGPCKMIAPILDEIADEYQGKLTVAKLNIDQNPGTAPKYG IRGIPTLLLFKNGEVAATKVGALSKGQLKEFLDANLAGTENLYF*QS). ARTD2_CAT_ was cloned into pNIC28-BSA4 (MHHHHHHSSGVDLGTENLYFQ*SM) while the other constructs (ARTD2_WGR+CAT_ and ARTD2_WGR_,) were cloned into a pNIC-ZB vector (MHHHHHHSSGVDNKFNKERRRARREIRHLPNLNREQRRAFIRSLRDDPSQSANLLAEAKKLNDAQPKGTENLYF*QS). The full-length ARTD2 construct was used as a template in cloning of the truncated fragments.

### Protein expression and purification

The expression vectors containing the individual genes were transformed into *E*. *coli BL*21 (DE3) competent cells. An overnight pre-culture supplemented with 50 μg/mL kanamycin was grown in LB broth medium at 37 °C. The pre-culture was used to inoculate terrific broth auto-induction media (Formedium) supplemented with 0.8% glycerol (w/v) and 50 μg/mL kanamycin. Notably in the expression of ARTD2_FL_ and ARTD2_WGR+CAT_ the expression media was supplemented with 10 mM benzamide, a general small ARTD inhibitor^33^, to overcome the toxic effect of the constructs on *E*. *coli*. The culture was incubated at 37 °C with shaking until the OD600 reached 1.2. The temperature was lowered to 18 °C and the incubation was continued for 22 hours. The cells were collected by centrifugation (4000 × g, at 4 °C for 30 minutes) and the cell pellet was suspended in 1.5 mL/g lysis buffer [50 mM Hepes (4-(2-hydroxyethyl)-1-piperazineethanesulfate), 500 mM NaCl, 10% glycerol, 10 mM imidazole, 0.5 mM TCEP (tris(2-carboxyethyl)phosphine), pH 7.5] and stored at −20 °C. The cell suspension was thawed at room temperature and supplemented with 0.1 mM Pefabloc (4-(2-Aminoethyl) benzenesulfonyl fluoride hydrochloride from Sigma-Aldrich). The cells were lysed by sonication and the cell debris cleared by centrifugation (30000 × g at 4 °C for 45 minutes). The supernatant was loaded onto 2 mL of IMAC resin (Qiagen). The column was washed with 30 mL of wash buffer 1 (50 mM Hepes, 500 mM NaCl, 10% glycerol, 10 mM imidazole, 0.5 mM TCEP, pH 7.5), followed by 30 mL wash buffer 2 (20 mM Hepes, 500 mM NaCl, 10% glycerol, 25 mM imidazole, 0.5 mM TCEP, pH 7.5). ARTD2 was eluted with 15 mL of elution buffer (50 mM Hepes, 250 mM NaCl, 10% glycerol, 200 mM imidazole, 0.5 mM TCEP, pH 7.5). The constructs containing the N-terminus or the WGR domain were purified by heparin chromatography with a gradient from 300 mM NaCl to 1500 mM NaCl. The fusion tags were cleaved with TEV-protease (1:30 TEV:ARTD2 molar ratio) at 4 °C for 24 hours. The protein was then loaded onto 1 mL HisTrap (GE Healthcare) and the flow through containing the cleaved proteins was collected, concentrated and further purified using Superdex 200 (for ARTD2_FL_, ARTD2_WGT+CAT_ and ARTD2_CAT_) and Superdex 75 (for ARTD2_N_, ARTD2_WGR_ and ARTD2_N+WGR_) (50 mM Hepes, 300 mM NaCl, 10% glycerol, 0.5 mM TCEP, pH 7.5). The purified protein was pooled, concentrated, flash frozen and stored in −70 °C.

### Activity assays

Assays were conducted as reported earlier^34^,^35^,^36^. The reactions were carried out in 96-well plates (Greiner BioOne U-shaped) at room temperature in 50 mM Tris-HCl pH 8.0, 10 μg/mL activated DNA (with the oligonucleotides at 0.5, 0.05, or 0.01 μM), 5 μM NAD^+^, 0.1 mg/ml BSA, 5 mM MgCl_2_ supplemented with 150 nM NaCl when mentioned. In most cases, the DNA and protein concentrations were varied to enable effective quantification of activation. At the completion of the reaction, 20 μL of 20% acetophenone in ethanol and 20 μL of 2 M KOH were added. The plate was incubated for 10 minutes and then 90 μL of formic acid was added. The fluorescence was read after 20 minutes of incubation using a Tecan Infinity M1000 at the excitation and emission wavelengths of 372 nm and 444 nm, respectively.

The fluorescence assay measures the consumption of NAD^+^ and we found that it correlates well with the analysis of the generated PAR chains with western blot method (Supplementary Fig. S2). ARTD2_FL_ (100 nM) was incubated with 100 nM Oligonucleotides 1, 2, 5, 6, 13, and 14 for 10 minutes at RT in the reaction buffer (50 mM Tris pH 8.0, 10 mM NAD^+^, and 10 mM MgCl_2_). Reactions were stopped by the addition of SDS-PAGE loading buffer, incubated at 95 °C for 5 minutes. The samples were resolved on 4–20% gradient gel (Bio-Rad) and blotted onto Nitrocellulose membrane (Bio-Rad). Blots were incubated with anti-PAR antibody (Trevigen) and then HRP conjugated mouse anti-goat antibody. PAR chains were visualized with an ECL plus reagent (Advansta).

### DNA binding assays

The DNA substrates were prepared by annealing the oligonucleotides (Fig. 1b; Supplementary Table S10) purchased from Integrated DNA Technologies in 10 mM Tris-HCl pH 8.0, 0.1 mM EDTA and 100 mM NaCl as described^37^. In the electrophoretic mobility shift assay (EMSA) the proteins were incubated with 1 μM oligonucleotides in a binding buffer (50 mM Hepes, 0.1 mM EDTA, 300 mM NaCl, 10% glycerol (V/V), 0.1 mM TCEP) for 30 minutes in an ice bath. The mixtures were resolved on 0.5% agarose gels in 0.5X Tris/Borate/EDTA buffer at +4 °C degrees, 80 volts for 80 minutes. The gels were stained using GelRed (Biotium) and visualized using a gel imaging system (Bio-Rad).

The fluorescence polarization assay (FP) was done in black 96-well U-bottom propylene plates (Greiner BioOne). Notably, the concentration of the protein samples were quantified prior to the measurements using calculated extinction coefficients and absorbance at 280 nm. The DNA amount was determined by the supplier (Integrated DNA Technologies) and the measured absorbance at 260 nm was found to agree with the calculated concentration. The reaction was carried out in 10 mM Hepes pH 8.0, 0.1 mM TCEP, 0.1 mM EDTA, 150 mM NaCl and 10% glycerol. DNA oligonucleotides 36–42 (Fig. 1b) were used. Specifically, 5 nM DNA was used and the protein concentration was titrated from 0–4 μM for ARTD2_FL_, and from 0–8 μM for the other constructs (Fig. 1a). The plates were incubated at 25 °C with shaking (300 rpm) in a PST-60 HL Plus shaker (Biosan) for 60 minutes. The fluorescence polarization was measured using a Tecan Infinite M1000 at the excitation and emission wavelengths of 475 and 520 nM, respectively. The experiment was done in triplicate and the measurements were fitted using Graphpad Prism version 5.04 for windows (GraphPad Software). Stoichiometry of ARTD2_FL_ DNA binding was measured with FP as described by Updegrove *et al*.^38^. We used DNA concentrations of 250 nM, which is significantly higher than the measured K_D_. The protein was titrated from 0–4 μM.

### Analytical HPLC for ARTD2 and DNA complexes

Analytical HPLC was done for ARTD2_FL_ and ARTD2_WGR+CAT_ in order to evaluate the complexes formed upon mixing ARTD2 and different oligonucleotides. The complexes were reconstituted at a ratio of 1:1 (Protein:DNA) at a concentration of 15 μM, and the samples (100 μL) were analyzed using a Superdex 200 10/300 GL column (20 mM Hepes pH 7.5, 300 mM NaCl, 5% glycerol). Using the elution volume of the *apo* enzymes and a comparison of a test separation of standard proteins in the column, the size of the complexes were estimated.

### Small-angle X-ray scattering studies

For the SAXS measurement of ARTD2_FL_ with DNA oligonucleotides 2 and 6, the protein-DNA-complex was reconstituted at a ratio of 1:1 and dialyzed into HPLC buffer (20 mM Hepes pH 7.5, 300 mM NaCl, 5% glycerol). The samples were run at a flow rate of 0.25 mL/min through a KW403-4F HPLC column (Shodex) connected to the online SAXS system on the BM29 beamline at ESRF (Grenoble, France). 100 μL of the samples were injected to the column and peaks in the scattering intensities for all frames were identified. Matching frames from individual peak were averaged. With the oligonucleotide 2 and 6, matching frames of the major peak corresponding to the 1:1 and 2:1 (protein:DNA) respectively were averaged. The scattering intensity corresponding to the complexes were processed using PRIMUS^39^. Distance distribution functions (Pr) and maximum distances (D_max_) were determined with GNOM^40^, and Porod volumes were determined with DATPOROD^41^. The *ab initio* model of the DNA complex was created with MONSA using data collected from the DNA, the protein and the complex^23^. Ten independent MONSA models without symmetry constraints were generated and 8 consistent models based on the NSD values were averaged using DAMAVER^42^.

For the truncated fragments and the DNA oligonucleotides, the SAXS measurements were done without chromatography at three different concentrations. The data were analyzed using the ATSAS suite^41^. Prior the experiments all the sample were centrifuged at 14,000 × g for 10 minutes. 50 μL of the individual protein sample was loaded into a sample capillary using a liquid handling and was exposed to X-rays. Scattering data were collected in multiple exposures (10 frames). Data processing and R_g_ determination were done with PRIMUS^39^. Distance distribution functions (Pr) and maximum distances (D_max_) were determined with GNOM^40^, and Porod volumes were determined with DATPOROD^41^. *Ab initio* molecules were generated with DAMMIF^43^ and 15 individual molecules were averaged with DAMAVER^42^. For rigid body modelling we used a homology model of the WGR domain and a crystal structure of the catalytic domain (PDB code 3KCZ) using the program bunch (Supplementary Fig. S4)^44^. The fit of the calculated scattering intensity of the crystal structure of the catalytic domain (PDB code 3KCZ) to the SAXS scattering intensity was analyzed using crysol^45^.

### Circular dichroism

CD spectra of ARTD2_FL_, ARTD2_WGR+CAT_ and ARTD2_N_ were recorded at 22 °C using Chiranscan^TM^ CD spectroscopy (Applied Photophysics Ltd.) equipped with a temperature-regulated sample chamber. A 1 mm path length quartz cuvette was used to obtain spectra in the far-UV region of 190–280 nm. The sample concentration was 0.04–0.06 mg/mL in 10 mM sodium phosphate pH 7.4 and 150 mM ammonium sulfate. The data were analyzed using the Pro-Data^TM^ Software suit (Applied Photophysics Ltd.).

## Additional Information

**How to cite this article**: Obaji, E. *et al*. Characterization of the DNA dependent activation of human ARTD2/PARP2. *Sci. Rep.* **6**, 34487; doi: 10.1038/srep34487 (2016).

## Supplementary Material

Supplementary Information

## Figures and Tables

**Table 1:** SAXS data analysis of the ARTD2, the truncated fragments, protein-DNA complexes and the oligonucleotides.

Samples	R_g_ (gnom) (Å)	R_g_ (guinier) (Å)	_maxq_R_g_	I_(0)_	Data points used
ARTD2_FL_ + OLIGO 2	41.86	41.52	1.19	111.40	9–50
ARTD2 + OLIGO 6	54.70	56.31	1.20	167.57	3–37
ARTD2_FL_	45.20	45.10	1.13	27.02	14–43
ARTD2_WGR+CAT_	40.71	40.70	1.15	42.91	8–48
ARTD2_N+WGR_	42.18	40.70	1.21	35.87	4–51
ARTD2_WGR_	20.30	20.20	1.05	15.48	4–113
ARTD2_CAT_	21.83	21.80	1.05	26.57	5–83
ARTD2_N_	28.10	28.10	1.07	8.75	5–70
Oligonucleotide 2	22.30	22.21	1.2	25.91	24–92
Oligonucleotide 6	14.93	14.71	1.09	13.96	16–140

**Table 2:** SAXS model parameters.

Samples	MW based on seq (KDa)	MW based porod volume (KDa)	Dmax (Å)	Porod volume (Å^3)^	NDS values (dammif)	NDS Values (MONSA)	chi^2^ (bunch)
ARTD2_FL_ + OLIGO 2	77.9	84.8	145.4	144084	—	0.56 ± 0.02	—
ARTD2 + OLIGO 6	138.8	182.9	187	311013	—	0.64 ± 0.03	—
ARTD2_FL_	66.3	72.1	159.2	103423	0.67 ± 0.03	—	2.14 ± 0.076
ARTD2_WGR+CAT_	58.7	57.4	142.5	97592	0.64 ± 0.04	—	—
ARTD2_N+WGR_	24.9	47.7	142.3	81153	0.69 ± 0.03	—	—
ARTD2_WGR_	17.4	17.1	71.2	29027	0.55 ± 0.02	—	—
ARTD2_CAT_	39.8	34.8	76.0	59190	0.60 ± 0.02	—	—
ARTD2_N_	7.8	9.3	98.0	15725	0.91 ± 0.06	—	—
Oligonucleotide 2	12.0	9.9	77.1	16969	0.61 ± 0.02	—	—
Oligonucleotide 6	6.0	5.7	51.5	9606	0.59 ± 0.03	—	—

*ARTD2_N+WGR_ is a dimeric protein. The monomeric form is 24.9 KDa and the data in the table represent the dimeric form of the protein.

**The scattering curve, Guinier plots and P(r) in the Supplementary Figs S3 and S9.

MW based porod volume were obtained by dividing the Porod volume estimated by AUTOPOROD by 1.7. Notably the porod volumes were obtained AUTOPOROD and the values obtained were divided by 1.7. 1.7 was taken to be a maximal ratio between the porod volume and the molecular weight^41^.

## References

[b1] Chambon P., Weill J. D. & Mandel P. Nicotinamide mononucleotide activation of new DNA-dependent polyadenylic acid synthesizing nuclear enzyme. Biochem. Biophys. Res. Commun. 11, 39–43 (1963). 14019961 10.1016/0006-291x(63)90024-x

[b2] Kutuzov M. M., Khodyreva S. N., Schreiber V. & Lavrik O. I. The role of PARP2 in DNA repair. Mol. Biol. (Mosk.) 48, 561–572 (2014). 25842842

[b3] Schreiber V., Dantzer F., Ame J.-C. & de Murcia G. Poly(ADP-ribose): novel functions for an old molecule. Nat. Rev. Mol. Cell Biol. 7, 517–528 (2006). 16829982 10.1038/nrm1963

[b4] Boehler C. . Poly(ADP-ribose) polymerase 3 (PARP3), a newcomer in cellular response to DNA damage and mitotic progression. Proc. Natl. Acad. Sci. USA 108, 2783–2788 (2011). 21270334 10.1073/pnas.1016574108 PMC3041075

[b5] Amé J.-C. . PARP-2, A Novel Mammalian DNA Damage-dependent Poly(ADP-ribose) Polymerase. J. Biol. Chem. 274, 17860–17868 (1999). 10364231 10.1074/jbc.274.25.17860

[b6] D’Amours D., Desnoyers S., D’Silva I. & Poirier G. G. Poly(ADP-ribosyl)ation reactions in the regulation of nuclear functions. Biochem. J. 342(Pt 2), 249–268 (1999). 10455009 PMC1220459

[b7] Hottiger M. O., Hassa P. O., Lüscher B., Schüler H. & Koch-Nolte F. Toward a unified nomenclature for mammalian ADP-ribosyltransferases. Trends Biochem. Sci. 35, 208–219 (2010). 20106667 10.1016/j.tibs.2009.12.003

[b8] Amé J.-C., Spenlehauer C. & de Murcia G. The PARP superfamily. Bioessays 26, 882–893 (2004). 15273990 10.1002/bies.20085

[b9] Otto H. . *In silico* characterization of the family of PARP-like poly(ADP-ribosyl)transferases (pARTs). BMC Genomics 6, 139 (2005). 16202152 10.1186/1471-2164-6-139 PMC1266365

[b10] De Vos M., Schreiber V. & Dantzer F. The diverse roles and clinical relevance of PARPs in DNA damage repair: Current state of the art. Biochem. Pharmacol. 84, 137–146 (2012). 22469522 10.1016/j.bcp.2012.03.018

[b11] Li M. & Yu X. The role of poly(ADP-ribosyl)ation in DNA damage response and cancer chemotherapy. Oncogene 34, 3349–3356 (2015). 25220415 10.1038/onc.2014.295 PMC4362780

[b12] Aguilar-Quesada R. . Modulation of transcription by PARP-1: consequences in carcinogenesis and inflammation. Curr. Med. Chem. 14, 1179–1187 (2007). 17504138 10.2174/092986707780597998

[b13] Masaoka A., Horton J. K., Beard W. A. & Wilson S. H. DNA polymerase beta and PARP activities in base excision repair in living cells. DNA Repair (Amst.) 8, 1290–1299 (2009). 19748837 10.1016/j.dnarep.2009.08.004 PMC2765039

[b14] Schreiber V. . Poly(ADP-ribose) polymerase-2 (PARP-2) is required for efficient base excision DNA repair in association with PARP-1 and XRCC1. J. Biol. Chem. 277, 23028–23036 (2002). 11948190 10.1074/jbc.M202390200

[b15] Pleschke J. M., Kleczkowska H. E., Strohm M. & Althaus F. R. Poly(ADP-ribose) binds to specific domains in DNA damage checkpoint proteins. J. Biol. Chem. 275, 40974–40980 (2000). 11016934 10.1074/jbc.M006520200

[b16] Kutuzov M. M. . Interaction of PARP-2 with DNA structures mimicking DNA repair intermediates and consequences on activity of base excision repair proteins. Biochimie 95, 1208–1215 (2013). 23357680 10.1016/j.biochi.2013.01.007

[b17] Langelier M.-F., Riccio A. A. & Pascal J. M. PARP-2 and PARP-3 are selectively activated by 5′ phosphorylated DNA breaks through an allosteric regulatory mechanism shared with PARP-1. Nucleic Acids Res. 42, 7762–7775 (2014). 24928857 10.1093/nar/gku474 PMC4081085

[b18] Kutuzov M. M. . Interaction of PARP-2 with AP site containing DNA. Biochimie 112, 10–19 (2015). 25724268 10.1016/j.biochi.2015.02.010

[b19] Léger K., Bär D., Savić N., Santoro R. & Hottiger M. O. ARTD2 activity is stimulated by RNA. Nucleic Acids Res. 42, 5072–5082 (2014). 24510188 10.1093/nar/gku131 PMC4005644

[b20] Riccio A. A., Cingolani G. & Pascal J. M. PARP-2 domain requirements for DNA damage-dependent activation and localization to sites of DNA damage. Nucleic Acids Res. 44, 1691–1702 (2016). 26704974 10.1093/nar/gkv1376 PMC4770219

[b21] Karlberg T., Hammarström M., Schütz P., Svensson L. & Schüler H. Crystal structure of the catalytic domain of human PARP2 in complex with PARP inhibitor ABT-888. Biochemistry 49, 1056–1058 (2010). 20092359 10.1021/bi902079y

[b22] Aoyagi-Scharber M. . Structural basis for the inhibition of poly(ADP-ribose) polymerases 1 and 2 by BMN 673, a potent inhibitor derived from dihydropyridophthalazinone. Acta Crystallogr. F. Struct. Biol. Commun. 70, 1143–1149 (2014). 25195882 10.1107/S2053230X14015088 PMC4157409

[b23] Svergun D. I. Restoring low resolution structure of biological macromolecules from solution scattering using simulated annealing. Biophys. J. 76, 2879–2886 (1999). 10354416 10.1016/S0006-3495(99)77443-6 PMC1300260

[b24] Dantzer F. . Base Excision Repair Is Impaired in Mammalian Cells Lacking Poly(ADP-ribose) Polymerase-1. Biochemistry 39, 7559–7569 (2000). 10858306 10.1021/bi0003442

[b25] Haince J.-F. . PARP1-dependent kinetics of recruitment of MRE11 and NBS1 proteins to multiple DNA damage sites. J. Biol. Chem. 283, 1197–1208 (2008). 18025084 10.1074/jbc.M706734200

[b26] Haince J.-F. . Ataxia telangiectasia mutated (ATM) signaling network is modulated by a novel poly(ADP-ribose)-dependent pathway in the early response to DNA-damaging agents. J. Biol. Chem. 282, 16441–16453 (2007). 17428792 10.1074/jbc.M608406200

[b27] Galande S. & Kohwi-Shigematsu T. Poly(ADP-ribose) polymerase and Ku autoantigen form a complex and synergistically bind to matrix attachment sequences. J. Biol. Chem. 274, 20521–20528 (1999). 10400681 10.1074/jbc.274.29.20521

[b28] Ali A. A. E. . The zinc-finger domains of PARP1 cooperate to recognize DNA strand breaks. Nat. Struct. Mol. Biol. 19, 685–692 (2012). 22683995 10.1038/nsmb.2335 PMC4826610

[b29] Haenni S. S. . Identification of lysines 36 and 37 of PARP-2 as targets for acetylation and auto-ADP-ribosylation. Int. J. Biochem. Cell. Biol. 40, 2274–2283 (2008). 18436469 10.1016/j.biocel.2008.03.008

[b30] Altmeyer M., Messner S., Hassa P. O., Fey M. & Hottiger M. O. Molecular mechanism of poly(ADP-ribosyl)ation by PARP1 and identification of lysine residues as ADP-ribose acceptor sites. Nucleic Acids Res. 37, 3723–3738 (2009). 19372272 10.1093/nar/gkp229 PMC2699514

[b31] Gagné J.-P. . Quantitative site-specific ADP-ribosylation profiling of DNA-dependent PARPs. DNA Repair (Amst.) 30, 68–79 (2015). 25800440 10.1016/j.dnarep.2015.02.004

[b32] Langelier M.-F., Planck J. L., Roy S. & Pascal J. M. Structural basis for DNA damage-dependent poly(ADP-ribosyl)ation by human PARP-1. Science 336, 728–732 (2012). 22582261 10.1126/science.1216338 PMC3532513

[b33] Banasik M., Komura H., Shimoyama M. & Ueda K. Specific inhibitors of poly(ADP-ribose) synthetase and mono(ADP-ribosyl)transferase. J. Biol. Chem. 267, 1569–1575 (1992). 1530940

[b34] Narwal M., Fallarero A., Vuorela P. & Lehtiö L. Homogeneous screening assay for human tankyrase. J. Biomol. Screen. 17, 593–604 (2012). 22357873 10.1177/1087057112436558

[b35] Narwal M., Venkannagari H. & Lehtiö L. Structural basis of selective inhibition of human tankyrases. J. Med. Chem. 55, 1360–1367 (2012). 22233320 10.1021/jm201510p

[b36] Haikarainen T. . para-Substituted 2-Phenyl-3,4-dihydroquinazolin-4-ones As Potent and Selective Tankyrase Inhibitors. ChemMedChem 8, 1978–1985 (2013). 24130191 10.1002/cmdc.201300337

[b37] Temsamani J., Kubert M. & Agrawal S. Sequence identity of the n-1 product of a synthetic oligonucleotide. Nucleic Acids Res. 23, 1841–1844 (1995). 7596808 10.1093/nar/23.11.1841 PMC306952

[b38] Updegrove T. B., Correia J. J., Chen Y., Terry C. & Wartell R. M. The stoichiometry of the Escherichia coli Hfq protein bound to RNA. RNA 17, 489–500 (2011). 21205841 10.1261/rna.2452111 PMC3039148

[b39] Konarev P. V., Volkov V. V., Sokolova A. V., Koch M. H. J. & Svergun D. I. PRIMUS: a Windows PC-based system for small-angle scattering data analysis. J. Appl. Crystallogr. 36, 1277–1282 (2003).

[b40] Svergun D. I. Determination of the regularization parameter in indirect-transform methods using perceptual criteria. J. Appl. Crystallogr. 25, 495–503 (1992).

[b41] Petoukhov M. V. . New developments in the ATSAS program package for small-angle scattering data analysis. J. Appl. Crystallogr. 45, 342–350 (2012). 25484842 10.1107/S0021889812007662 PMC4233345

[b42] Volkov V. V. & Svergun D. I. Uniqueness of ab initio shape determination in small-angle scattering. J. Appl. Cryst. 36, 860–864 (2003). 10.1107/S0021889809000338 PMC5023043 27630371

[b43] Franke D. & Svergun D. I. DAMMIF, a program for rapid ab-initio shape determination in small-angle scattering. J. Appl. Crystallogr. 42, 342–346 (2009). 27630371 10.1107/S0021889809000338 PMC5023043

[b44] Petoukhov M. V. & Svergun D. I. Global Rigid Body Modeling of Macromolecular Complexes against Small-Angle Scattering Data. Biophys. J. 89, 1237–1250 (2005). 15923225 10.1529/biophysj.105.064154 PMC1366608

[b45] Svergun D., Barberato C. & Koch M. H. J. CRYSOL - a Program to Evaluate X-ray Solution Scattering of Biological Macromolecules from Atomic Coordinates. J. Appl. Cryst. 28, 768–773 (1995).

