# Supplementary material for: Characterization of the DNA dependent activation of human ARTD2/PARP2
Source: Sci Rep. 2016 Oct 6;6:34487. doi: 10.1038/srep34487 (PMC5052650; doi:10.1038/srep34487)
Supplement: Supplementary Information [file srep34487-s1.pdf]

Supplementary Information

Characterization of DNA dependent activation of human ARTD2/PARP2

Ezeogo Obaji Teemu Haikarainen and Lari Lehtiö*

Faculty of Biochemistry and Molecular Medicine & Biocenter Oulu, University of Oulu, Oulu, Finland

* Corresponding author: Lari Lehtiö, Lari.Lehtio@oulu.fi

CONTENT

Supplementary Figure S1: SDS-PAGE analysis of ARTD2 constructs used in this study.

Supplementary Figure S2: Fluorescence activity assay of ARTD2 and Western blot assay.

Supplementary Figure S3: SAXS Guinier plots.

Supplementary Figure S4. Rigid body modeling.

Supplementary Figure S5: Scattering intensity and *Ab initio* models of ARTD2 constructs.

Supplementary Figure S6: HPLC and Rg analysis of $ARTD2_{FL}$ and $ARTD2_{WGR+CAT}$ as well as their complexes with DNA oligonucleotides.

Supplementary Figure S7: Sequence alignment of human ARTD2 isoform 1 and isoform 2.

Supplementary Figure S8: Activation of $ARTD2_{FL}$ by RNA in comparison to activating single stranded DNA.

Supplementary Figure S9: Small angle X-ray scattering analysis of the oligonucleotides 2 and 6.

Supplementary Table S10: Sequences and structures of the oligonucleotides used in this study.

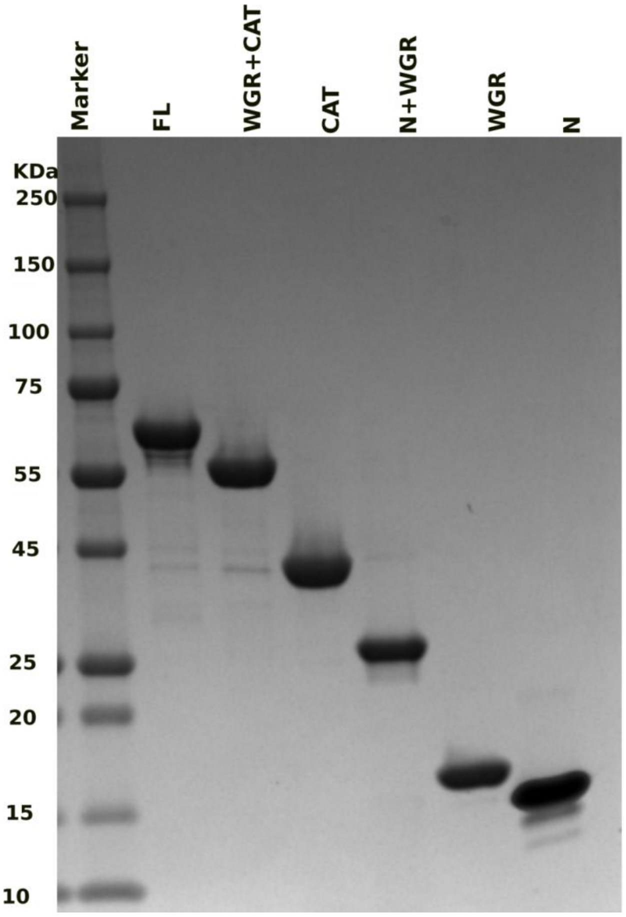

Supplementary Figure S1. SDS-PAGE analysis of ARTD2 constructs that was used in this study. Representing the $ARTD2_{FL}$, $ARTD2_{WGR+CAT}$, $ARTD2_{CAT}$, $ARTD2_{N+WGR,}$ $ARTD2_{WGR}$ and $ARTD2_{N.}$

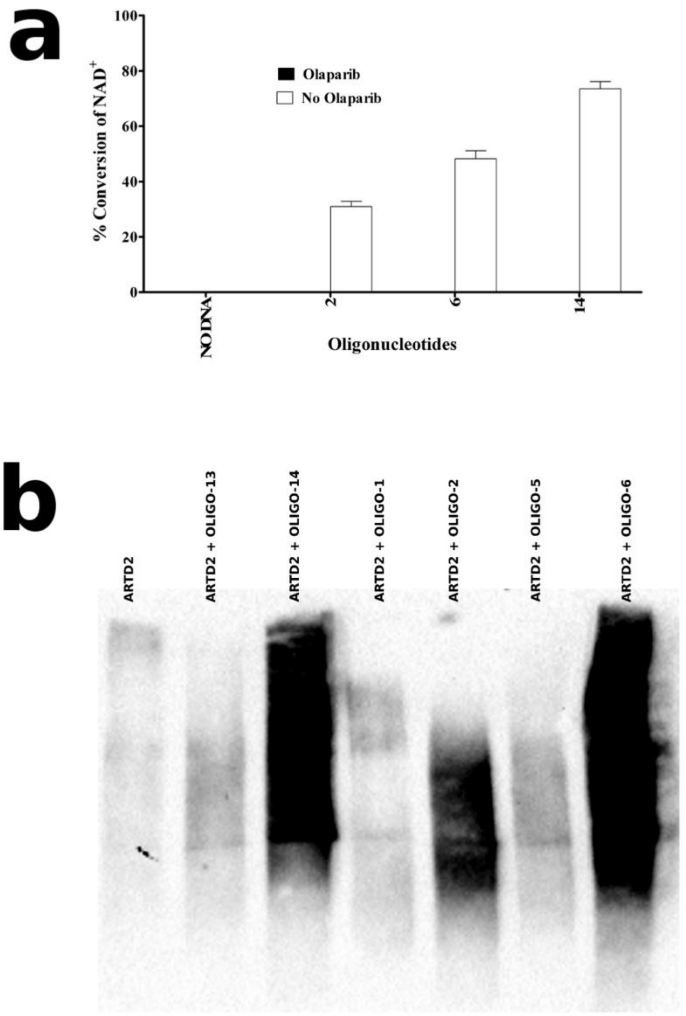

Supplementary Figure S2. Comparison of activity assays of ARTD2. (a) The activation of ARTD2 by oligonucleotide 2, 6 and 14 was measured in the presence of 1 µM olaparib. This confirms that the measured activities were based on ARTD2 catalytic function and not any contamination like an NADase. (b) Western blot assay to analyze how NAD^+ hydrolysis of ARTD2 correlates with ADP-ribosylation. The assay was done using oligonucleotide 1, 2, 5, 6, 13, and 14. The result is inline with the NAD^+ hydrolysis measured using fluorescence assay where oligonucleotides 14 and 6 were observed to have higher activation on ARTD2 than 2. Also little or no activity was observed with non phosphorylated oligonucleotides (13, 1, and 5).

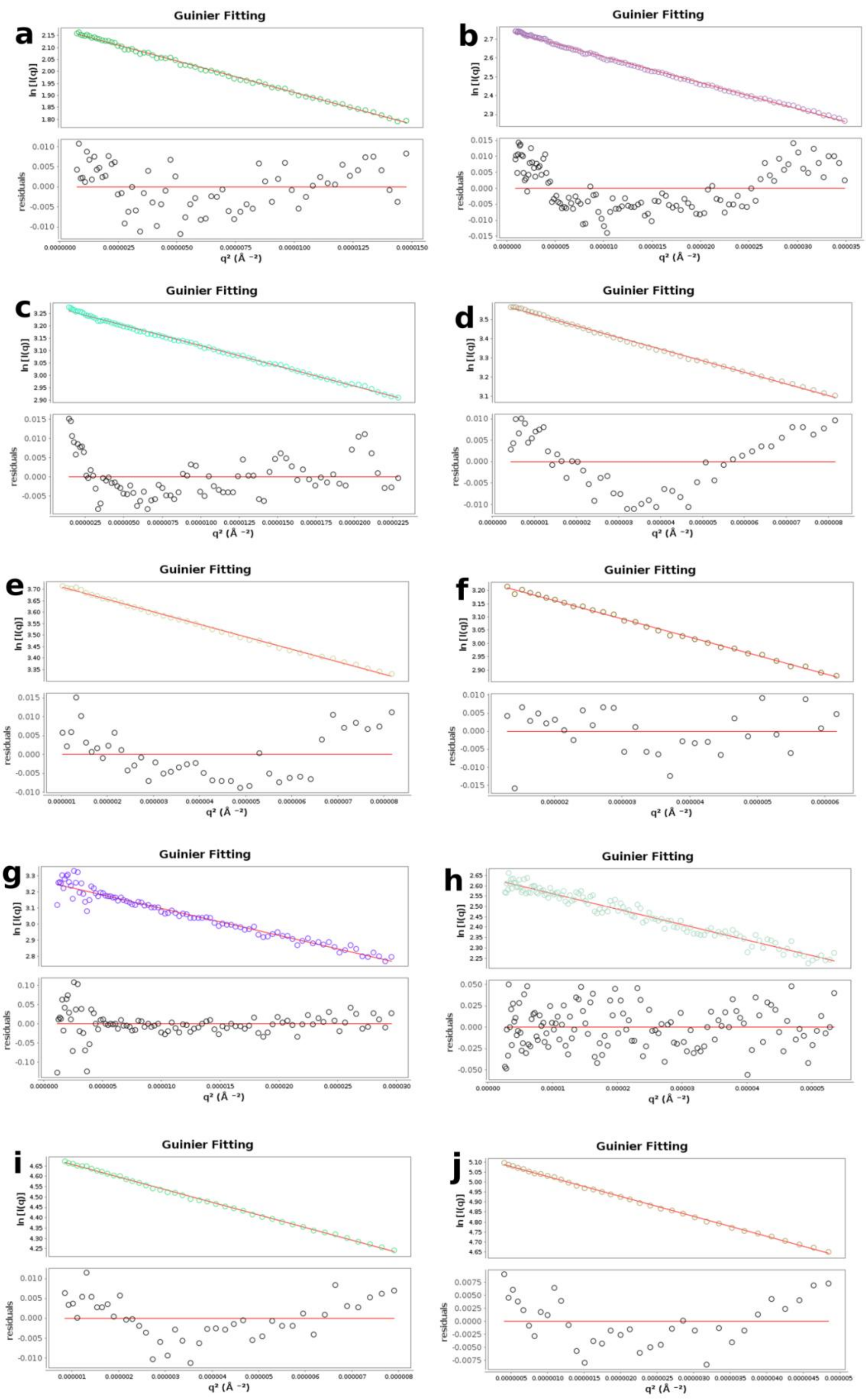

Supplementary Figure S3. The Guinier plots. Separate Guinier plots for each of the samples (a) ARTD2$_{N,}$ (b) ARTD2$_{WGR}$, (c) ARTD2$_{CAT}$, (d) ARTD2$_{N+WGR}$, (e) ARTD2$_{WGR+CAT}$, (f) ARTD2$_{FL}$, (g) Oligonucleotide 2, (h) Oligonucleotide 6, (i) ARTD2$_{FL}$+OLIGO2, and (j) ARTD2$_{FL}$+OLIGO2 respectively), indicating linear fits in the low angle regions.

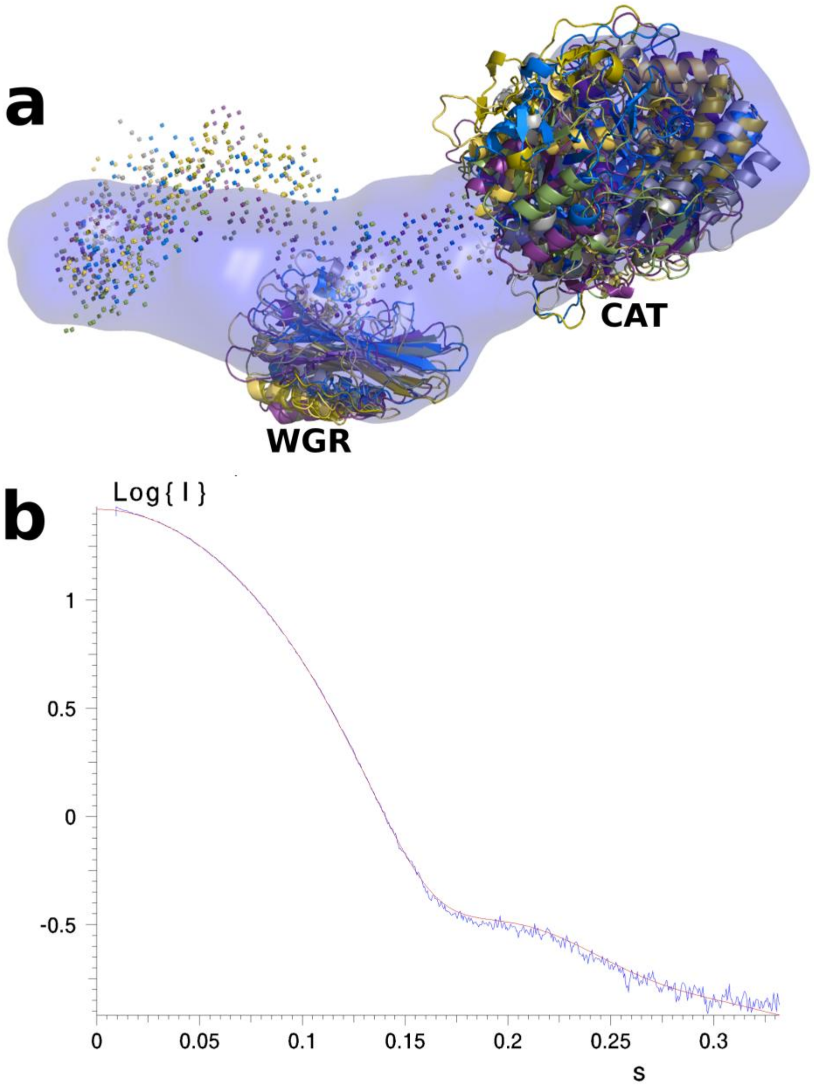

Supplementary Figure S4. Rigid body modeling of ARTD2$_{FL}$ and comparison of the calculated ARTD$_{CAT}$ crystal structure (PDB code 3KCZ) scattering intensity with the SAXS scattering intensity. (a) 6 bunch generated models with Chi^2 = 2.14 ± 0.076. (b) Crysol analysis of the crystal structure of ARTD2$_{CAT}$ with SAXS scattering data with Chi^2 = 1.26.

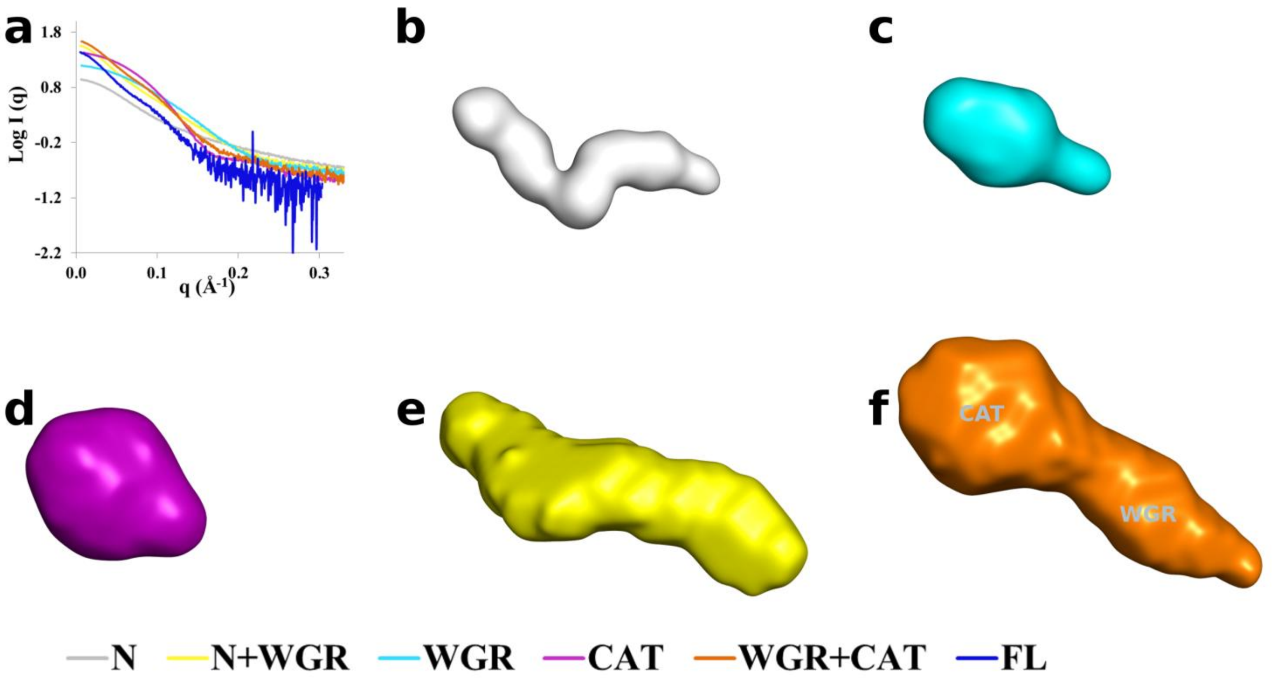

Supplementary Figure S5. Scattering intensity and *Ab initio* models of ARTD2 constructs. (a) The scattering profile of $ARTD2_{FL}$ and the truncated fragments. (b-f) *Ab initio* models of $ARTD2_{N,}$ $ARTD2_{WGR}$, $ARTD2_{CAT}$, $ARTD2_{N+WGR}$ and $ARTD2_{WGR+CAT,}$ respectively. 15 independent runs were done for each and models were averaged using damaver. Pictures (b-f) were made with Pymol (Schrödinger).

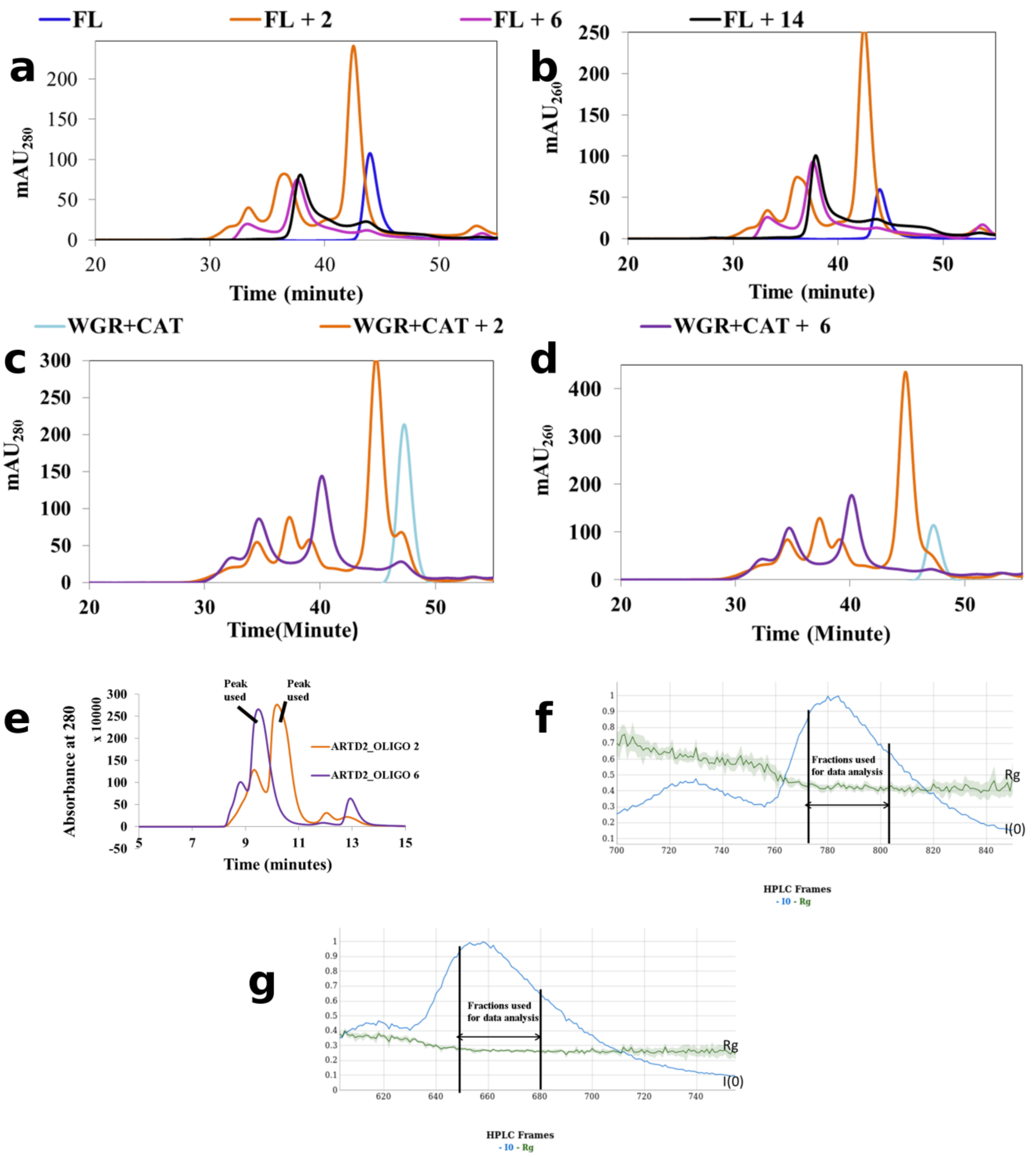

Supplementary Figure S6. HPLC analysis of $ARTD2_{FL}$ and $ARTD2_{WGR+CAT}$ as well as their complexes with DNA oligonucleotides 2, 6 and 14 using superdex 200 10/300 GL and SEC-SAXS profile and Rg profile across the target peaks from the SEC-SAXS. Analysis of $ARTD2_{FL}$ and its complexes with DNA oligonucleotides 2, 6 and 14 were carried out at (a) 280 nm and (b) 260 nm wavelengths. Analysis of $ARTD2_{WGR+CAT}$ and its complexes with DNA oligonucleotide 2 and 6 at (c) 280 nm and (d) 260 nm wavelengths. Overall, the blue represent the monomeric form of $ARTD2_{FL}$, yellow line represent the ARTD2 in complex with oligonucleotide 2, purple line represent the ARTD2 in complex with oligonucleotide 6, the black line represent the ARTD2 in complex with oligonucleotide 14, cyan line represent the $ARTD2_{WGR+CAT}$. Upon DNA binding, ARTD2 forms different oligomeric states (monomeric, dimeric and higher oligomeric states). (e) SEC-SAXS profile of ARTD2 in complex with oligonucleotide 2 (yellow) and 6 (purple). (f-g) Rg (green line) and scattering intensity (blue line) profile of ARTD2 in complex with oligonucleotide 2 (f) and 6 (g).

```
Iso_1          1 MAARRRRSTGGGRARALNESKRVNNGNTAPEDSSPAKKTRRCQRQESKKMPVAGGKANKD
Iso_2          1 MAARRRRSTGGGRARALNESKRVNNGNTAPEDSSPAKKTRRCQRQESKKMPVAGGKANKD
                 ************************************************************

Iso_1         61 RTEDKQDGMPGRSWASKRVSESVKALLLKGKAPVDPECTAKVGKAHVYCEGNDVYDVMLN
Iso_2         61 RTEDKQD-------------ESVKALLLKGKAPVDPECTAKVGKAHVYCEGNDVYDVMLN
                 *******             ****************************************

Iso_1        121 QTNLQFNNNKYYLIQLLEDDAQRNFSVWMRWGRVGKMGQHSLVACSGNLNKAKEIFQKKF
Iso_2        108 QTNLQFNNNKYYLIQLLEDDAQRNFSVWMRWGRVGKMGQHSLVACSGNLNKAKEIFQKKF
                 ************************************************************

Iso_1        181 LDKTKNNWEDREKFEKVPGKYDMLQMDYATNTQDEEETKKEESLKSPLKPESQLDLRVQE
Iso_2        168 LDKTKNNWEDREKFEKVPGKYDMLQMDYATNTQDEEETKKEESLKSPLKPESQLDLRVQE
                 ************************************************************

Iso_1        241 LIKLICNVQAMEEMMMEMKYNTKKAPLGKLTVAQIKAGYQSLKKIEDCIRAGQHGRALME
Iso_2        228 LIKLICNVQAMEEMMMEMKYNTKKAPLGKLTVAQIKAGYQSLKKIEDCIRAGQHGRALME
                 ************************************************************

Iso_1        301 ACNEFYTRIPHDFGLRTPPLIRTQKELSEKIQLLEALGDIEIAIKLVKTELQSPEHPLDQ
Iso_2        288 ACNEFYTRIPHDFGLRTPPLIRTQKELSEKIQLLEALGDIEIAIKLVKTELQSPEHPLDQ
                 ************************************************************

Iso_1        361 HYRNLHCALRPLDHESYEFKVISQYLQSTHAPTHSDYTMTLLDLFEVEKDGEKEAFREDL
Iso_2        348 HYRNLHCALRPLDHESYEFKVISQYLQSTHAPTHSDYTMTLLDLFEVEKDGEKEAFREDL
                 ************************************************************

Iso_1        421 HNRMLLWHGSRMSNWVGILSHGLRIAPPEAPITGYMFGKGIYFADMSSKSANYCFASRLK
Iso_2        408 HNRMLLWHGSRMSNWVGILSHGLRIAPPEAPITGYMFGKGIYFADMSSKSANYCFASRLK
                 ************************************************************
```

Supplementary Figure S7. Sequence alignment of human ARTD2 isoform 1 (Uniprot Id. NP-005475) and isoform 2 (Uniprot Id. NP-001036083).

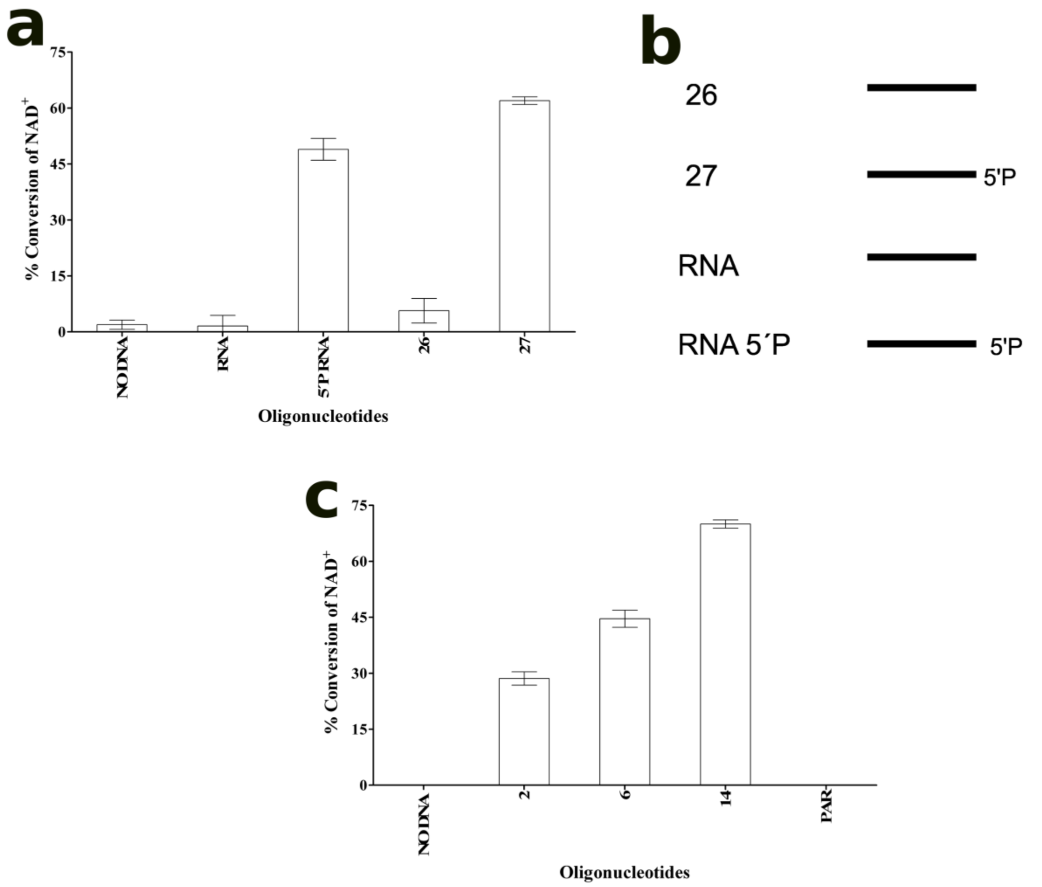

Supplementary Figure S8. Activation of ARTD2_{FL} by RNA in comparison to activating single stranded DNA. (a) Activity assay of ARTD2 in the presence of single stranded RNA and DNA with and without 5´phosphate and without DNA. (b) Schematic representation of the RNA and DNA used (see Supplementary Figure S2 for the sequence). (c) Activation of ARTD2 by PAR chain (Trevigen #4336-100-01) in comparison to Oligonucleotide 2, 6, and 14.

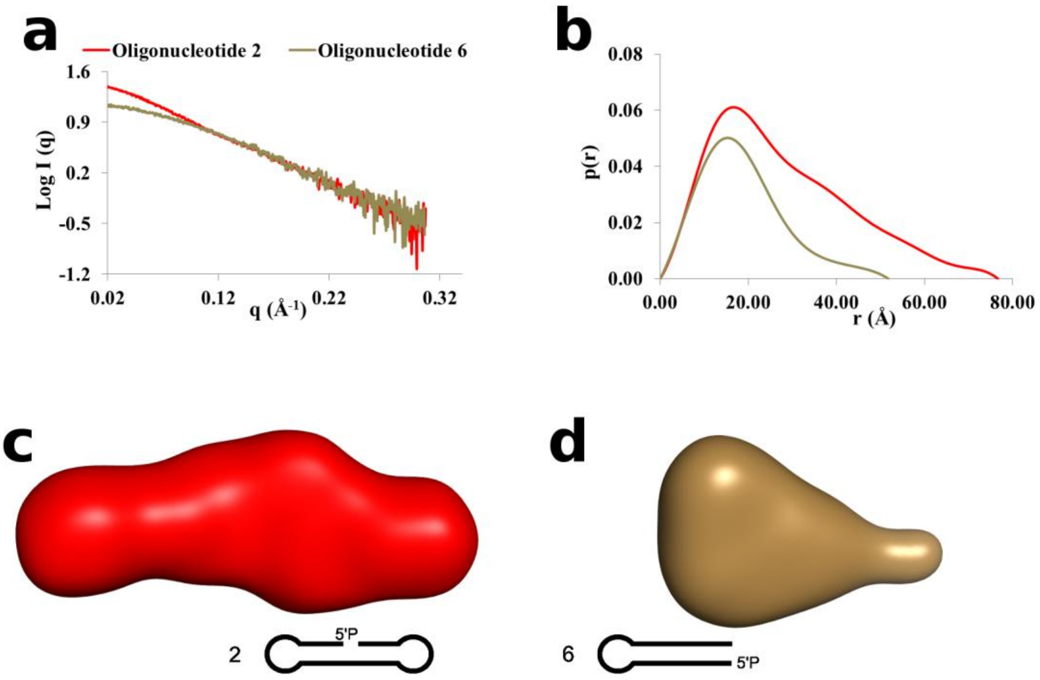

Supplementary Figure S9. (a) The scattering profiles of oligonucleotides 2 and 6 (b) The P(r) distribution profile of oligonucleotides 2 and 6 (c-d) *ab initio* model of oligonucleotides 2 and 6, respectively. 15 independent runs were done for each and models were averaged using damaver. Pictures (b-f) were made with Pymol (Schrödinger).

Supplementary Table S10. Sequences and structures of the oligonucleotides used in the study.

**1**	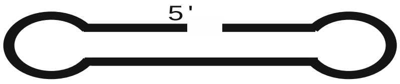<br><br>**5´ GGAAGTTCTTTTGAACTTCCTCGAAGCTTTTGCTTCGA 3´**
**2**	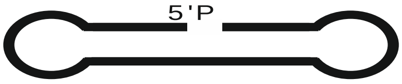<br><br>**5'-P GGAAGTTCTTTTGAACTTCCTCGAAGCTTTTGCTTCGA 3´**
**3**	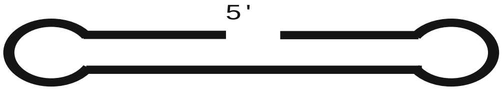<br><br>**5´ GCTGAGCTTCTGGTGAAGCTCAGCTCGCGGCAGCTGGTGCTGC CGCGA 3´**
**4**	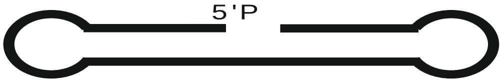<br><br>**5´-P GCTGAGCTTCTGGTGAAGCTCAGCTCGCGGCAGCTGGTGCTGC CGCGA 3´**
**5**	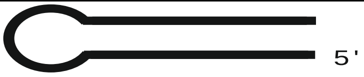<br><br>**5´ GGA AGT TCT TTT GAA CTT CC 3'**
**6**	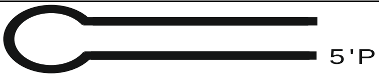<br><br>**5´-P GGA AGT TCT TTT GAA CTT CC 3'**
**7**	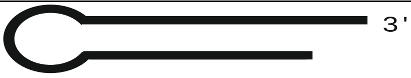<br><br>**5´ GGA AGT TCT TTT GAA CTT CCA 3´**
**8**	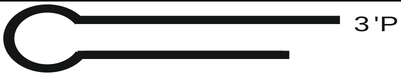<br><br>**5´ GGA AGT TCT TTT GAA CTT CCA 3´-P**
**9**	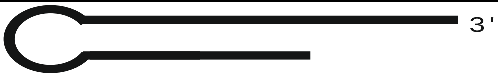<br><br>**5´ GGA AGT TCT TTT GAA CTT CCGG A 3´**
**10**	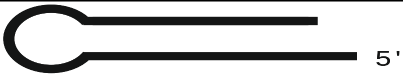<br><br>**5' ACC AAG TTC TTT TGA ACT TGG 3'**
**11**	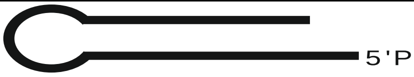<br><br>**5´-P ACC AAG TTC TTT TGA ACT TGG 3'**
**12**	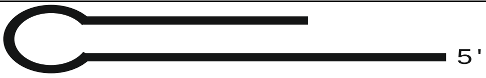<br><br>**5' AGGCC AAG TTC TTT TGA ACT TGG 3´**
**13**	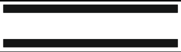

	**5´ GCCTAT AGG C 3´**
**14**	5'P 5'P<br>**5´P- GCCTAT AGG C 3´**
**15**	**5´ GCC TAG CTA TAG CTA GGC 3´**
**16**	5'P 5'P<br>**5´P- GCC TAG CTA TAG CTA GGC 3´**
**17**	**5´ GCC TTA GCG CGT ATA CGC GCT AAG GC 3**
**18**	5'P 5'P<br>**5´P- GCC TTA GCG CGT ATA CGC GCT AAG GC 3´**
**19**	**5'- CCAGGACCAGGGCGCAGATCACCTTGTTCTCCA -3'**
**20**	5'P 5'P<br>**5'P- CCAGGACCAGGGCGCAGATCACCTTGTTCTCCA -3'**
**21**	**5'- GCC ACT AGT CTC GCA GTT AGC GCG TAT ACG CGC TAA CTG CGA GAC TAG TGG C -3'**
**22**	5'P 5'P<br>**5'P- GCC ACT AGT CTC GCA GTT AGC GCG TAT ACG CGC TAA CTG CGA GAC TAG TGG C -3'**
**23**	**5´P- GCT CTA GTG TAG CAT GAG CCT TAG CGC GTT TTC GCG CTA AGG CTC ATG CTA CAC TAG AGC 3´**
**24**	5'P 5'P<br>**5´P- GCT CTA GTG TAG CAT GAG CCT TAG CGC GTT TTC GCG CTA AGG CTC ATG CTA CAC TAG AGC 3´**
**25**	5'P 5'P<br>**5´P-GAT CGT TGC CGT ATA GCT TAG TGT AAG CAT GAG CCT TAG CGC GTA TAC GCG CTA AGG CTC ATG CTT ACA CTA AGC TAT ACG GCA ACG ATC 3´**
**26**	

	**5´ GCCTAT AGG C 3´**
**27**	5'P<br>**5´P GCCTAT AGG C 3´**
**28**	**5´ GCC TAG CTA TAG CTA GGC 3´**
**29**	5'P<br>**5´P GCC TAG CTA TAG CTA GGC 3´**
**30**	**5'- GCC ACT AGT CTC GCA GTT AGC GCG TAT ACG CGC TAA CTG CGA GAC TAG TGG C -3'**
**31**	5'P<br>**5'P- GCC ACT AGT CTC GCA GTT AGC GCG TAT ACG CGC TAA CTG CGA GAC TAG TGG C -3'**
**32**	**5´ GCT GGT ATG TAG GAA GAG CCT TAG CAA GCC 3´**
**33**	5'P<br>**5´P GCT GGT ATG TAG GAA GAG CCT TAG CAA GCC 3´**
**34**	**5´ TTT TTT TTT TTT TTT TTT TTT TTT TTT TTT 3`**
**35**	5'P<br>**5´P TTT TTT TTT TTT TTT TTT TTT TTT TTT TTT 3`**
**36**	**Equivalent of oligonucleotide 14 but with fluorescein tag**
**37**	**Equivalent of oligonucleotide 27 but with fluorescein tag**
**38**	**Equivalent of oligonucleotide 18 but with fluorescein tag**
**37**	**Equivalent of oligonucleotide 19 but with fluorescein tag**
**40**	**Equivalent of oligonucleotide 20 but with fluorescein tag**
**41**	**Equivalent of oligonucleotide 2 but with fluorescein tag**
**42**	**Equivalent of oligonucleotide 6 but with fluorescein tag**
